# Single-cell and spatial transcriptomic investigation reveals the spatiotemporal specificity of the beta-defensin gene family during mouse sperm maturation

**DOI:** 10.1186/s12964-024-01637-3

**Published:** 2024-05-14

**Authors:** Guoliang Zhang, Yuanchao Sun, Minkai Guan, Mengmeng Liu, Shiduo Sun

**Affiliations:** 1 Qingdao Agricultural University, Qingdao, China; 2 Qingdao University, Qingdao, China; 3 Northwest A&F University, Yangling, China

**Keywords:** Spermatogenesis, Spatiotemporal specificity, Epididymis, Beta-defensin genes

## Abstract

Low sperm motility is a significant contributor to male infertility. beta-defensins have been implicated in host defence and the acquisition of sperm motility; however, the regulatory mechanisms governing their gene expression patterns and functions remain poorly understood. In this study, we performed single-cell RNA and spatial transcriptome sequencing to investigate the cellular composition of testicular and epididymal tissues and examined their gene expression characteristics. In the epididymis, we found that epididymal epithelial cells display a region specificity of gene expression in different epididymal segments, including the beta-defensin family genes. In particular, *Defb15*, *Defb18*, *Defb20*, *Defb25* and *Defb48* are specific to the caput; *Defb22*, *Defb23* and *Defb26* to the corpus; *Defb2* and *Defb9* to the cauda of the epididymis. To confirm this, we performed mRNA fluorescence in situ hybridisation (FISH) targeting certain exon region of beta-defensin genes, and found some of their expression matched the sequencing results and displayed a close connection with epididimosome marker gene *Cd63*. In addition, we paid attention to the Sertoli cells and Leydig cells in the testis, along with fibroblasts and smooth muscle cells in the epididymis, by demonstrating their gene expression profile and spatial information. Our study provides a single-cell and spatial landscape for analysing the gene expression characteristics of testicular and epididymal environments and has important implications for the study of spermatogenesis and sperm maturation.

**Supplementary Information:**

The online version contains supplementary material available at 10.1186/s12964-024-01637-3.

## Introduction

In recent years, male fertility has decreased significantly, and one of the important reasons for male infertility is low sperm motility. The sperm cells within the testes of mammals are immature and incapable of fertilisation [1]. Acquisition of sperm motility and maturation occurs in the epididymis [2–4], making it important to understand the epididymal microenvironment of sperm maturation. The epididymis contains a long, highly coiled tube that connects the testes to the vas deferens. It can be divided into three parts: the caput, corpus, and cauda, which exhibit highly segment-specific characteristics [5]. There are four main types of epithelial cells within the epididymal duct: narrow, clear, principal, and basal cells [6]. Principal cells are the major type of epididymal epithelial cells that are distributed in all three segments and constitute up to 80% of the tubular epithelium. The principal cells in the caput region demonstrate heightened protein synthesis and secretion activity, housing numerous secretory organelles including the endoplasmic reticulum, Golgi apparatus, and secretory granules, which exhibit active exocytosis activity [7–10]. Principal cells in the caudal region mainly play a role in ingesting the contents of the epididymal lumen through a receptor-mediated mechanism and reabsorbing the epididymal fluid together with clear cells [11]. Clear cells were extensively distributed throughout the caput and corpus regions of the epididymis, with a notable concentration observed in the cauda region. The apex region of clear cells contains rich endocytotic structures, demonstrating a strong endocytosis capacity, and is responsible for regulating lumen pH [5]. Narrow cells are mainly distributed in the initial segment of the epididymis and their function remains unclear.

The liquid microenvironment in the epididymal duct provides a platform for sperm motility and maturation. The ionic components of epididymal fluid are unique and show segmental specificity. The epididymal fluid is acidic to ensure that the sperm remains in a resting state and contains abundant protein components, such as albumin and lectins [12, 13]. In addition, epididymal epithelial cells regulate sperm state through ionic components. For example, Ca^2+^ secreted by epididymal epithelial cells regulates the activity of proton pumps, thereby regulating the epididymal pH [5]. Simultaneously, Ca^2+^ in the epididymal fluid directly regulates sperm function through the cation channel of sperm (CatSper) in the flagella [12].

Numerous studies have shown that many innate immunity secretory genes, including beta-defensin genes, are expressed in epididymal epithelial cells and play important roles in sperm motility acquisition and maturation [14–20]. beta-defensins are a class of cationic polypeptides rich in arginine and have a broad spectrum of antimicrobial activity [21, 22]. Many defensin genes are expressed in epididymal epithelial cells, and the defensin proteins encoded by these genes are believed to act synergistically in the epididymal fluid, thereby jointly realising the protection, maturation, and fertilisation abilities of sperm in the epididymal duct [23, 24]. In vitro experiments have shown that recombinant defensins possess dose-dependent antibacterial activity and that these antimicrobial peptides bind to the surface of sperm to protect them from bacterial or viral attacks within the genital tract [15]. The expression of beta-defensin family genes demonstrated segmental specificity in the epididymis. Defensin beta 12 (*Defb12*), *Defb15,* and *Defb17* are reported to be expressed in the caput of the epididymis, while *Defb22* and *28* are expressed in the corpus and cauda segments of the epididymis [14, 25–28]. Numerous studies have shown that the knockout of neighbouring beta-defensin family genes on chromosome 8 in mice hinders calcium regulation and the acrosomal reaction of sperm [29, 30]. *Bin1b* is a rat epididymis-specific beta-defensin gene with human genetic homology whose protein can bind to the sperm head in different ways, promoting the motility of immature sperm by activating the L-type Ca^2+^ channel of sperm [15]. These above studies have demonstrated that beta-defensin family genes play a crucial role in sperm maturation and even fertility, but little is known about the underlying mechanisms that regulating their expression and function in the epididymis.

The epididymal fluid is mainly composed of secretions of epididymal epithelial cells, which release synthesised ions and proteins into the epididymal fluid via apical plasma secretion [31, 32]. Studies have shown that principal and clear cells promote sperm maturation by releasing epididymosomes to transport synthetic proteins and small non-coding RNAs (sncRNAs) [33, 34]. With the application of widely used single-cell RNA sequencing (scRNA-seq) technology, cell types and their gene expression profiles at single-cell resolution can be browsed within the testis and epididymis [35–40]. Single-cell data have their own disadvantages because during the preparation of single-cell samples, it needs to dissociate the testicular or epididymal tissues into a single-cell suspension, losing the spatial location information of identified cell clusters. Therefore, the combination of single-cell and spatial transcriptome sequencing technology will be more conducive for studying the cellular environment and molecular mechanisms of spermatogenesis and sperm maturation.

In this study, we performed single-cell and spatial transcriptomic sequencing to identify the cellular environment of testicular and epididymal tissues and investigated their gene expression profiles. In addition, we profiled different segments of the mouse epididymis and described their gene expression characteristics, as well as the segmental specificity expression of beta-defensin family genes. Our data will aid in comprehending the underlying mechanisms of beta-defensin gene expression patterns, offering a comprehensive perspective on both spermatogenesis and sperm maturation.

Result.

### Cell compositions and their gene expression profile in mouse testis

**Fig.1:**
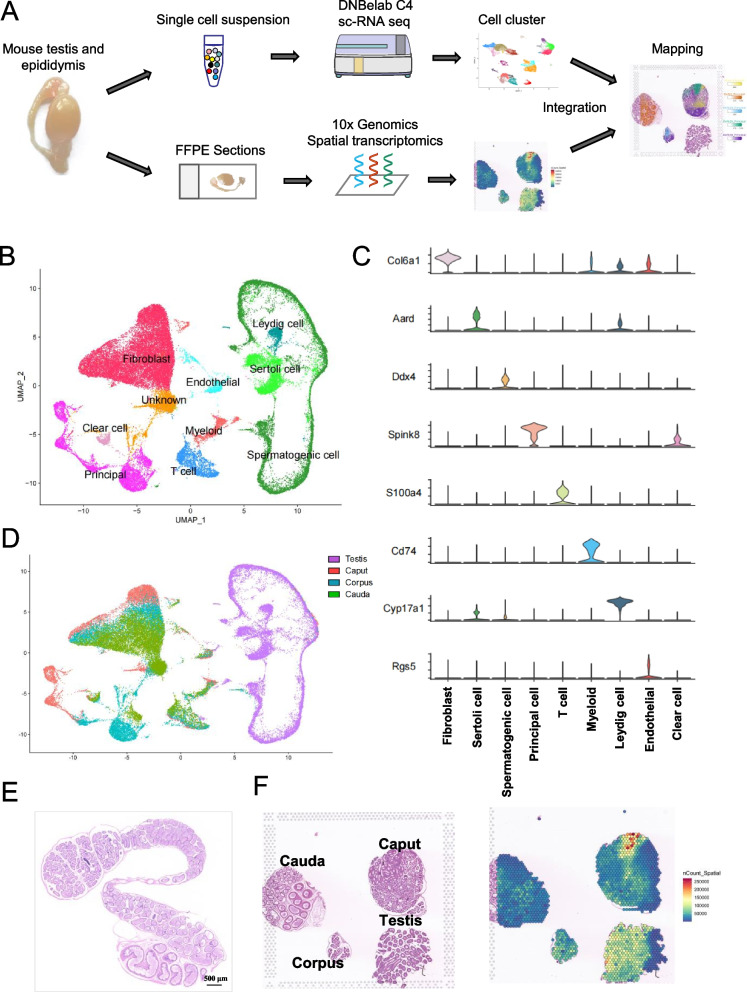
Single-cell and spatial transcriptomics analysis of mouse testicular and epididymal tissues**. A** Graphic landscape of this study design. Testicular and epididymal tissues underwent scRNA-seq analysis using the DNBSEQ C4 platform. Both longitudinal and crosscut sections of these tissues were then subjected to visium spatial transcriptomics using the 10 × Genomics platform. **B** Uniform manifold approximation and projection (UMAP) of 45,852 cells from 4 samples. **C** UMAP plots of all cells from 4 orig.ident samples. **D** Violin plots of related gene expression across 9 recognised cell clusters. **E** Representative H&E-stained section of the longitudinal section of mouse epididymis. Scale bar: 500 μm. **F** H&E staining of tissue sections (left) and unbiased clustering of spatial spots (right) in the transverse section of testicular and epididymal tissues

With the exception of spermatogenic cells, other residents in the testis and epididymis play crucial roles in sperm development. To examine their gene expression profiles and spatial information, we conducted single-cell RNA sequencing (scRNA-seq) using the DNBSQ platform for four samples. Additionally, spatial analysis was performed on testicular and epididymal samples using the 10X Genomics platform. The four scRNA-seq samples included one testicular and three epididymal samples (Fig. 1A). After removing the low-quality cells, 45,852 cells were obtained for subsequent analysis using Seurat V4 [41]. According to the marker gene expression in each cell group (Fig. 1B and C), the obtained cells were mainly classified into 11 cell clusters, among which testicular cells contained Leydig cells, Sertoli cells, and spermatogenic cells, which were mainly composed of principal cells, clear cells, fibroblasts, smooth muscle cells, and immune cells (Fig. 1D, Fig. S1A). Sperm develop in the testes and undergo motility and maturation in the epididymis, highlighting the importance of gaining a comprehensive understanding of the cell subpopulations within both organs. The mammalian epididymis contains a long, highly coiled tube, where sperm acquire motility and maturation for fertilisation (Fig. 1E). To obtain the landscape of spatial gene expression profiles of the mouse testis and epididymis, we performed visible spatial transcriptome sequencing on the crosscut (Fig. 1F) and longitudinal sections (described below) and predicted the location of single-cell clusters on the spatial samples using Cell2location package.

**Fig. 2:**
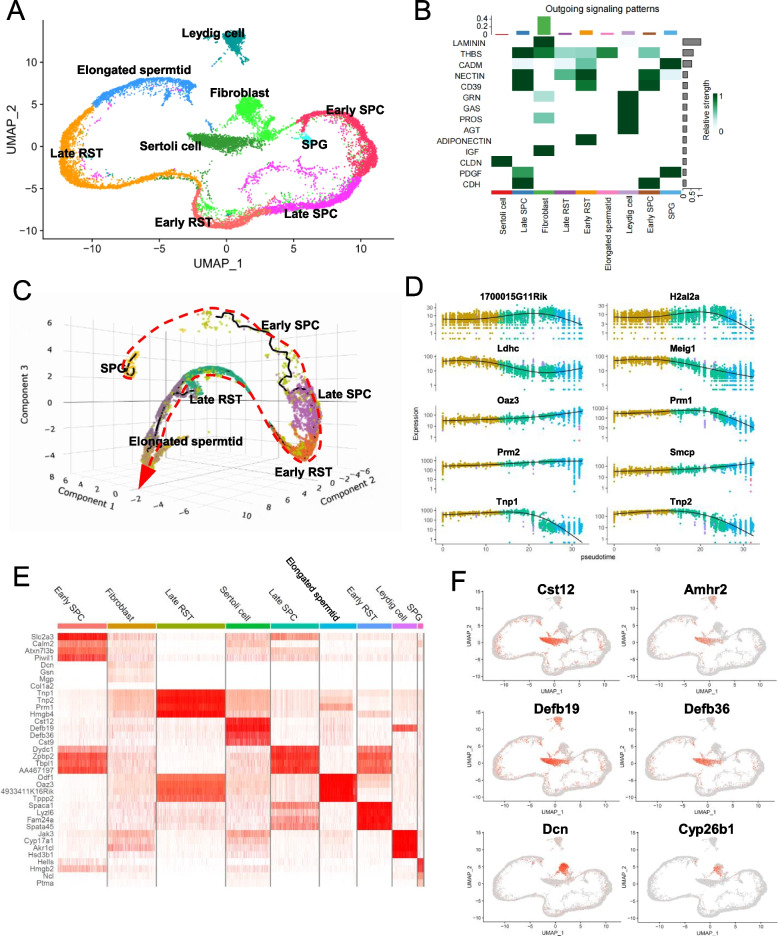
Single-cell gene expression profiles of testicular cells. **A** UMAP of testicular cell subsets, including Leydig cell, Sertoli cell, Fibroblast, SPG, Early SPC, Late SPC, Early RST, Late RST, and Elongated spermatid, each cluster was shown in different colour. **B** Outgoing signalling patterns of each testicular cell cluster, the bars shown right represent the relative strength of each signaling in different cell clusters, while the top represent the communication probability of signaling patterns from each cell cluster. **C** Single-cell 3D trajectories of spermatogenic cells from single-cell RNA sequencing data by Monocle 3. **D** Plots of Top 10 genes that contribute to the trajectories. E) DoHeatmap of top 4 genes in each cell cluster. **F** Feature plots of associated gene expression across stromal subsets in mouse testis

First, to further explore the cellular environment of spermatogenesis in the testis, we extracted testicular cells and set the resolution to 1.2 to reclassify them. We obtained nine subsets based on marker gene expression (Fig. 2A, Fig. S1B, and C, Table S1). These cell sets included Sertoli cells, Leydig cells, fibroblasts, spermatogonial cells (SPG), early spermatocyte cells (Early SPC), Late SPC, early round spermatids (Early RST), Late RST and Elongated spermatids. We used CellChat to evaluate the cell–cell communication among these subpopulations of testicular cells, and showed that the output signalling pattern from each cell cluster is closely related to its function; for example, Sertoli cells display positive CLDN signals, which are connected with tight junctions, and SPG is closely related to the maintenance of stem cell characteristics (Fig. 2B). The UMAP plot data showed that spermatogenic cells were distributed as long lines from early stage SPG to late RST in the order of sperm development. We also constructed single-cell 3D trajectories using Monocle 3 and found that the spermatogenic cells were ordered by the pseudotime of natural spermatogenesis (Fig. 2C). The top 10 genes that contributed to the trajectories were mainly involved in the transformation from late RST to Elongated spermatids (Fig. 2D). Interestingly, serving as the support and nutrient supply centre for spermatogenic cells, the Sertoli cell highly expressed cystatin 9 (*Cst9*), *Cst12,* and beta-defensin genes (*Defb19*, *Defb36*), associated with antifungal innate immune response (Fig. 2E and F). Furthermore, Defb33 was active in Late RST (Fig. S2A). Given the recognised role of beta-defensins in sperm maturation, further exploration of beta-defensin genes expression in testis is warranted. In addition to the single-cell sequencing data, the expression of *Defb19*, *Defb33* and *Defb36* were also found in the spatial samples (Fig. S2B-D). To confirm this, we performed RNA fluorescence in situ hybridisation (FISH) targeting certain exon regions of *Defb19* and found that mRNAof *Defb19* is abundant in Sertoli cells and early stage spermatogenic cells within seminiferous tubule (Fig. S2E), revealing that beta-defensin maybe also act during spermatogenesis.

### Epididymal epithelium cells display a region-specific gene expression profile

**Fig. 3:**
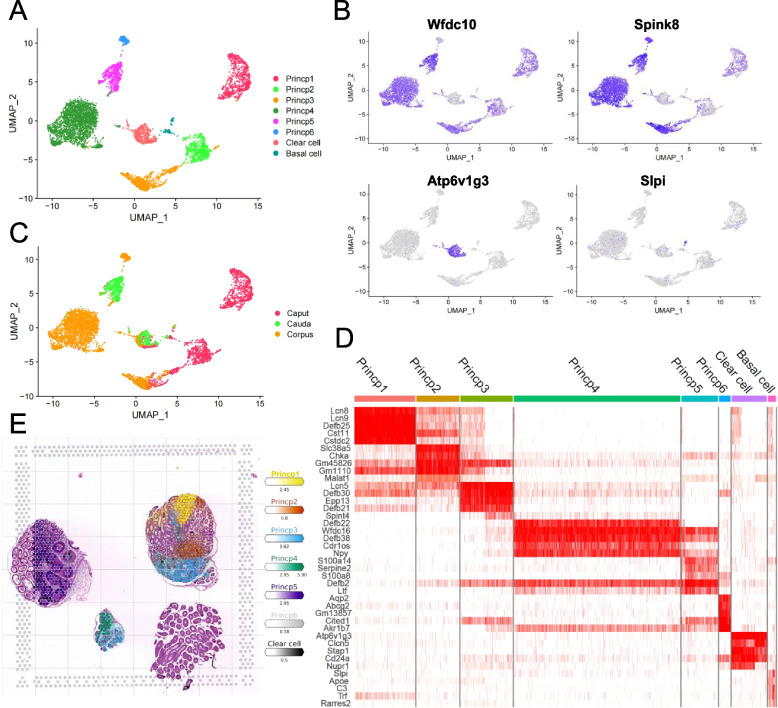
Cell subsets of epididymal epithelium and their gene expression profile. **A** UMAP of epididymal epithelium cell subsets, including Clear cell, Basal cell, and 6 subsets of principal cells, each cell cluster was shown in different colour. **B** Feature plots of epididymal epithelium associated gene expression across these subsets. **C** UMAP plots of epididymal epithelium cells from 3 samples, including the caput, corpus, and cauda region of epididymis. **D** DoHeatmap of top 5 genes in each cell cluster of epididymal epithelium. **E** Mapping spatial data with scRNA-Seq cell type annotations by using Cell2location

Epididymal cells comprise both epithelial and stromal cells. Relevant functional studies have confirmed that these cells have different spatial distributions and play crucial roles in sperm maturation [11, 42]. To thoroughly investigate the in detail information of epididymal epithelial cells at the single-cell level, we extracted epididymal epithelial cells, set the resolution to 1.2 for subdivision, and obtained clear cells, basal cells, and six subsets of principal cells according to the expression of their marker genes (Fig. 3A and B, Table S2). Interestingly, the six principal cell subsets exhibited distinct gene expression patterns. For example, lipocalin 8 (*Lcn8*), *Lcn9*, *Defb25* and solute carrier family 38 member 5 (*Slc38a5*) are specifically expressed in the caput region of the epididymis. *Defb22* and *S100a4* were expressed in the corpus and cauda, respectively (Fig. 3C and D). scRNA-seq data have the resolution of a single cell but lose spatial information. While spatial transcriptome data offer a spatial perspective on gene expression, the spot's diameter is significantly larger than that of a single cell. Therefore, integrating the analyses of both can harness the advantages of each more effectively. Therefore, we applied the Cell2location to map the location of cell clusters from the scRNA-seq data [43, 44]. Through integrated analysis and the mapping function of Cell2location, we obtained the spatial distribution of the epididymal epithelium, among which the six principal cell subsets displayed high region specificity in different segments (Fig. 3E, Fig. S3).

**Fig. 4:**
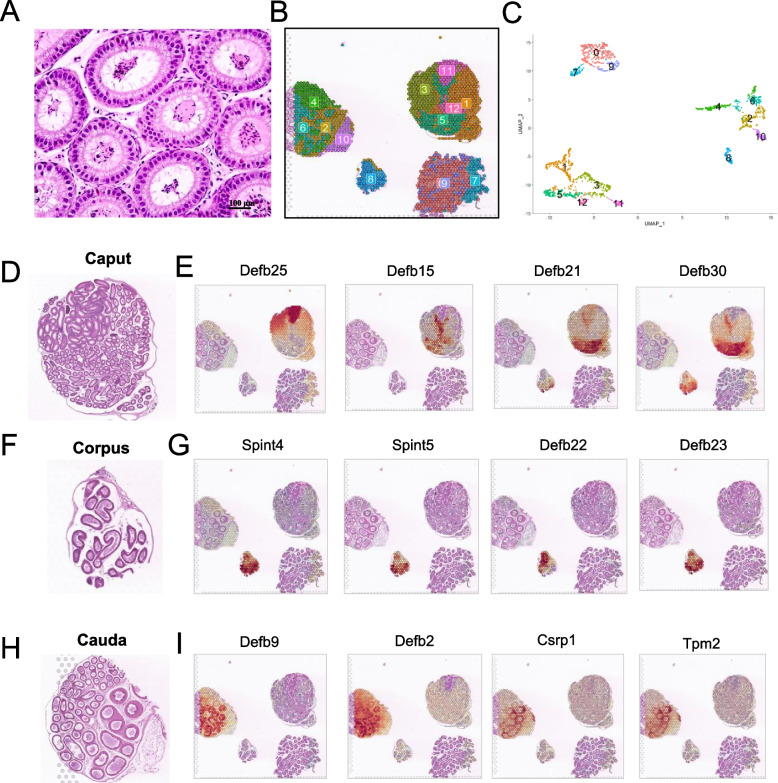
Mouse epididymal epithelium showed region specificity in gene expression profile. **A** Representative H&E-stained section of the caput epididymides. Scale bar: 50 μm. **B** Unbiased clustering of spatial spots in the crosscut spatial sample. **C** UMAP plot of spot transcriptome clusters in the spatial sample. **D** Enlarged view of caput epididymides in Fig. 1F. **E** Spatial gene expression features of *Defb25*, *Defb15*, *Defb21,* and *Defb30* in crosscut spatial sample. **F** Enlarged view of corpus epididymides. **G** Spatial gene expression features of *Spint4*, *Spint5*, *Defb22,* and *Defb23* in crosscut spatial sample of the epididymis. **H** Enlarged view of cauda epididymides in Fig. 1F. **I** Spatial gene expression features of *Defb2*, *Defb9*, *Csrp1,* and *Tpm2* in the crosscut spatial sample

Principal cells accounted for approximately 80% of the epithelial cells within the epididymal duct (Fig. 4A) and displayed a certain continuity within the entire epididymal duct. In the crosscut spatial sample of the epididymis, the spot cluster distribution showed a certain corresponded with the structural characteristics of the epididymis (4B and 4C). The scRNA-seq data demonstrated that some members of the beta-defensin gene family display regional specificity in the epididymis while lacking a spatial panoramic view. Hence, we initially investigated the spatial expression of the entire beta-defensin family genes on crosscut samples. We observed that, while *Defb25*, *Defb15*, *Defb21, Defb30* and *Defb20* (described below) were expressed in caput, their expression located in significantly different region of the caput epididymis (Fig. 4D and E). In the corpus epididymis, it displayed a specific expression of *Defb22*, *Defb23*, serine protease inhibitor, Kunitz type 4 (*Spint4*), and *Spint5* (Fig. 4F and G). Moreover, the caudal epididymis mainly expressed *Defb2*, *Defb9*, cysteine and glycine-rich protein 1 (*Csrp1*) as well as tropomyosin 2 and beta (*Tpm2*) (Fig. 4H and I). Therefore, these results revealed the region specificity in epididymis, especially beta-defensins family genes, indicating that different beta-defensins expressed in different regions might be involved in subregional or coordinated regulation on sperm maturation or motility acquisition, which need further investigation. The expression of other beta-defensin genes was also analysed (Fig. S4).

**Fig. 5:**
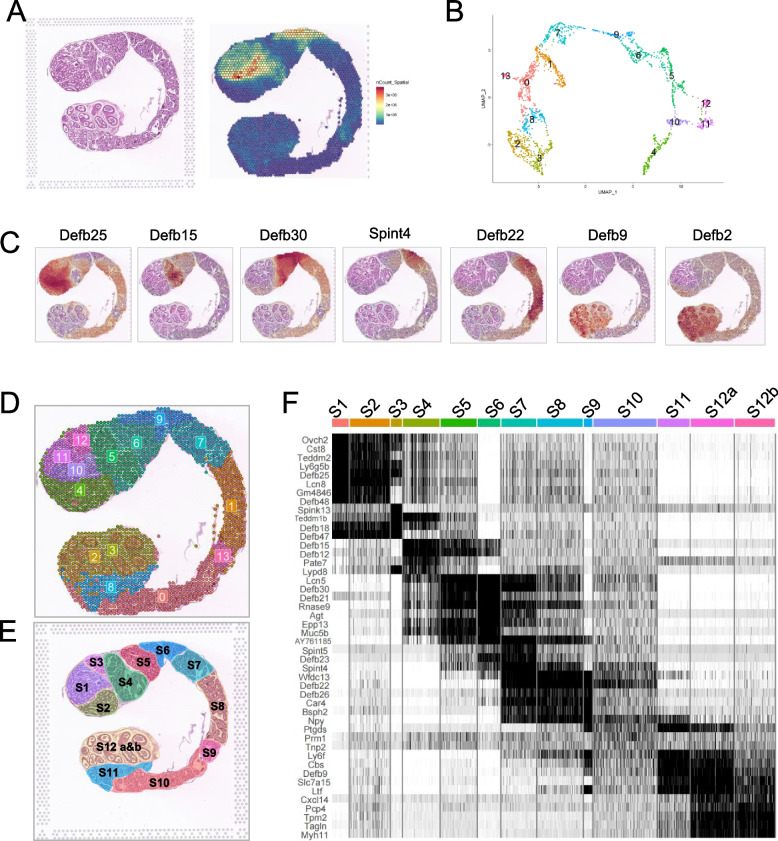
The longitudinal spatial sample provided a more intuitive view of gene expression with region specificity. **A** H&E staining of tissue sections (left) and nCount_Spatial features (right) of the longitudinal spatial sample, the “nCount_Spatial features” represents the sum of all genes expressed in each spot. **B** Unbiased clustering of spatial spots in the longitudinal spatial sample. **C** Spatial gene expression features of *Defb18*, *Defb48*, *Defb25*, *Defb15*, *Defb21*, *Defb30*, *Defb29*, *Defb23*, *Defb26*, *Defb22*, *Defb2,* and *Defb9* in longitudinal spatial sample. **D** The spatial distribution of spot clusters in Fig. 5B on the longitudinal spatial sample. **E** The longitudinal spatial sample was segmented into 12 sections based on spot cluster distribution and structural characteristics. F) Heatmap of top 4 gene in each segment

Second, to confirm the results of the crosscut spatial sampling of the epididymis, we conducted a longitudinal spatial sampling. In the longitudinal spatial sample, gene expression was quite active in the initial segment of the epididymis (Fig. 5A). We clustered the spots to examine their gene expression profiles (Fig. 5B). Subsequent to that, we identified the beta-defensin genes of interest and specifically observed the expression of *Defb18, Defb48, Defb25, Defb15* in the caput region; *Defb21*, *Defb30* and *Defb29* in the region where the caput and corpus connect; *Defb26*, *Defb22* and *Defb23* in the corpus; *Defb2* and *Defb9* in the caudal region of epididymis. This pattern aligns with the trends observed in the crosscut spatial sample. (Fig. 5C, Fig. S5). Mapping of spatial data with scRNA-Seq cell type annotations in the longitudinal spatial sample was also conducted by Cell2location (S6A, B, and C), which showed a similar trend to the crosscut spatial sample. Based on the distribution of the spot clusters (Fig. 5D) and the structural characteristics of the epididymis, we divided the longitudinal spatial sample of the epididymis into 12 segments (Fig. 5E). As segment 12 contains the internal and external regions of the epididymal duct, segment 12 is divided into S12a and S12b. Using the function of identifying Seurat markers, we conducted gene expression analysis on 12 segments, listing the top five genes and all gene expression profiles (Fig. 5F). We also analysed the gene expression in each segment (Table S3), which will provide a basis for follow-up studies on epididymal region-specific expression.

### mRNA of the beta-defensin genes showed a close connection to epididimosome

**Fig. 6:**
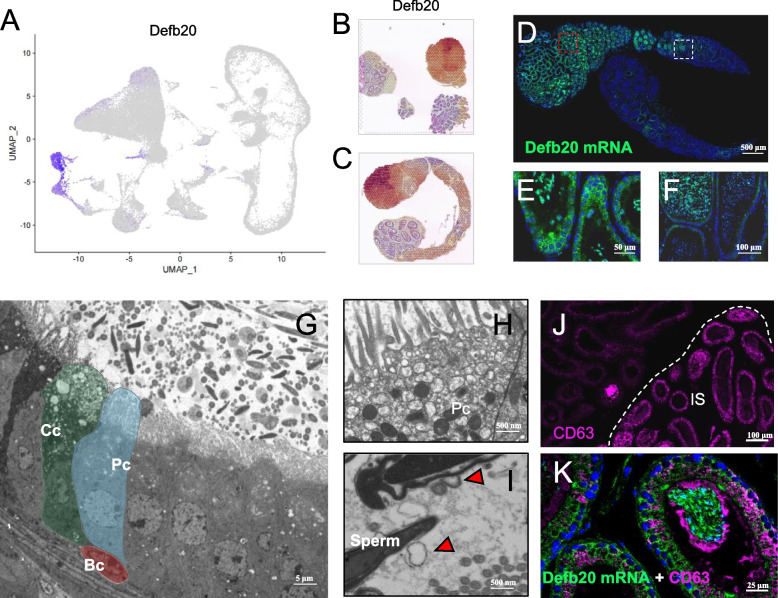
mRNA of *Defb20* exhibit on sperm in the epididymal lumen. **A** Feature plot of *Defb20* in all cell types. **B** Spatial gene expression features of *Defb20* in crosscut spatial sample. **C** Spatial gene expression features of *Defb20* in longitudinal spatial sample. **D** RNA fluorescence in situ hybridisation (FISH) staining of *Defb20* with mouse epididymis. Scale bar: 500 μm. **E** Enlarged view of Fig. 6D (red frame), showing mRNA of *Defb20* to be positive in principal cell and sperm. Scale bar: 50 μm. **F** Enlarged view of Fig.. 6D (white frame), showing positive of mRNA of *Defb20* on sperm to be tapered off for disappearing, in corpus region of epididymis. Scale bar: 50 μm. **G** Electron microscope images of epididymal epithelium. Cc: clear cell, Pc: principal cell. Scale bar: 5 μm. **H** Electron microscope images of principal cell with apocrine activity. Scale bar: 500 nm. **I** Electron microscope images of epididymal lumen showing epididymosomes around the surface of sperm. Scale bar: 500 nm. **J** Representative IF staining of CD63 in epididymis, IS: initial segment. Scale bar: 50 μm. **K** Representative IF staining of CD63 and FISH staining of *Defb20* mRNA in the caput region of epididymis. Scale bar: 50 μm

The present study shows that the expression of beta-defensin family genes displayed spatiotemporal specificity in the mouse epididymis. Other studies also demonstrated that beta-defensin genes are active in the epididymis and play important roles in sperm maturation [21, 28, 45, 46]. *Defb20* is specifically expressed in the caput region of mouse epididymis and may be involved in sperm maturation [45]. Our data also confirmed this observation (Fig. 6A). In both crosscut and longitudinal spatial samples, *Defb20* was expressed in the caput region (Fig.  6B and C). To confirm this, we performed RNA FISH targeting certain exon regions of *Defb20*, *Defb30,* and *Defb9* in mouse epididymis FFPE sections and found that the mRNA of *Defb20* is specifically located in the principal cells of the caput region (Fig. 6D). Interestingly, *Defb20* mRNA was not only present in the principal cells but was also abundant in sperm head, which gradually disappeared near the corpus region (Fig. 6E and F). Proteins and small noncoding RNA (sncRNAs) synthesised by principal cells are secreted into the epididymal lumen via apocrine secretion. Exosomes produced by epididymal epithelial cells act as transport carriers to transmit proteins and scnRNAs to the sperm and directly participate in the regulation of sperm maturation [7, 47, 48], which is in agreement with our electron microscopy observations (Fig. 6G, H, and I), providing a way to communicate between somatic cells and sperm in the epididymis. As an important marker gene associated with exosomes, CD63 was active in the initial segment (IS) region (Fig. 6J, Fig. S7A). Through FISH co-staining of *Defb20* mRNA with the exosomal protein CD63, we found that CD63 and *Defb20* mRNA display a close association (Fig. 6K), revealing that *Defb20* mRNA may be transported by epididymosomes to the sperm; however, further confirmation is required. In addition, the mRNA of *Defb30* and *Defb9* were located in the corpus and cauda regions, respectively (Fig. S7B and C), which was in accordance with the single-cell and spatial sequencing data. To confirm this at protein level, we performed immunofluorescence staining with DEFB121 (*Defb21* homolog in human) antibody, and found it’s protein expression pattern in epididymis showed similarity with it’s mRNA at single-cell and spatial level (Figs. 4E and 5C, Fig. S7D-G). These findings suggest that beta-defensin gene mRNA is synthesised not only in the epididymal epithelium but also may be transported to the sperm surface through epididymosomes. The presence of beta-defensins’ mRNA in sperm head and its impact on sperm maturation is worth further investigation.

### Stromal cells and their spatial information in the mouse epididymis

**Fig. 7:**
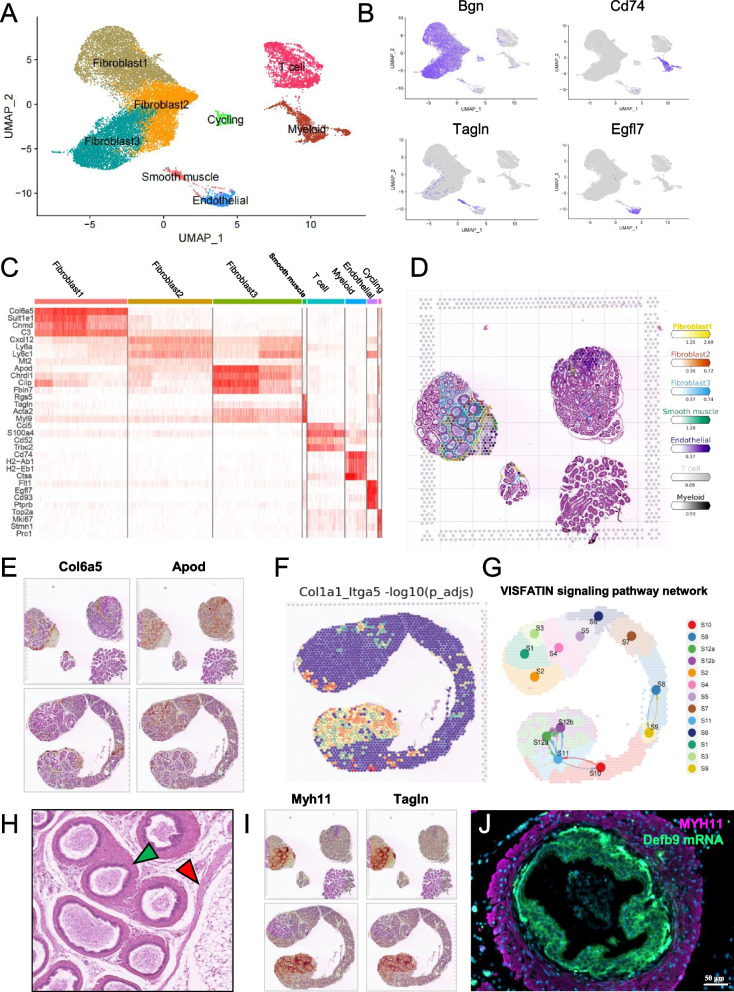
Stromal cells and their gene expression profile in mouse epididymis. **A** UMAP of stromal cell subsets, including Smooth muscle, Endothelial, Myeloid, T cell, Cycling and 3 subsets of fibroblast; each cell cluster is shown in different colour. **B** Feature plots of associated gene expression across these subsets. **C** DoHeatmap of top 5 genes in each cell cluster of stromal cell subsets. **D** Mapping spatial data with scRNA-Seq cell type annotations by using Cell2location. **E** Spatial gene expression features of *Col6a5* and *Apod* in the crosscut and longitudinal spatial sample. **F** Distribution of Col1a1_Igta5 receptor–ligand interactions in the longitudinal sptial sample. **G** VISFATIN signalling pathway in the longitudinal sptial sample. **H** Enlarged view of cauda epididymides in Fig. 1F (blue frame), showing smooth muscle (green arrow) around ductus epididymidis and fibrous boundary (red arrow) in the cauda region of epididymis. **I** Spatial gene expression features of *Myh11* in the crosscut and longitudinal spatial sample. J) Representative IF staining of MYH11 and *Defb9* mRNA FISH staining in the posterior region of caudal epididymis. Scale bar: 50 μm

In addition to the main epididymal epithelial cells, the mouse epididymis contains other cell types, including fibroblasts, endothelial cells, smooth muscle cells, and immune cells. These cell types and their associations with epididymal epithelial cells are also important. We extracted stromal cells and reclassified them according to their gene expression profiles to obtain smooth muscle, Endothelial, T cell, myeloid, cyclin, and three fibroblast subsets (Fig. 7A, B, and C, Table S4). In terms of the tissue structure, there were fewer fibroblasts in the caput and corpus regions, which acted mainly as separation compartments. Abundant fibroblasts and smooth muscle cells were observed in the caudal epididymis. In this study, we predicted the spatial localisation of stromal cell subsets in scRNA-seq data and found that the distribution of these cell clusters was similar to the actual situation. In addition, we obtained detailed localisation information for each cell subtype, such as the distribution of the three fibroblast subsets in the epididymis (Figs. 7D and S6B). Furthermore, the mapping data indicated that Fibroblast1 that highly expresses collagen, type VI, and alpha 5 (*Col6a5*), may act as the compartment or boundary of different segments of the epididymis, while Fibroblast3 that highly expresses apolipoprotein D (*Apod*) and chordin-like 1 (*Chrdl1*), is distributed in both the caput and cauda regions of the epididymis, indicating active signal transduction (Fig. 7E, Fig. S8A and B). We utilised stlearn for the spatial communication analysis of our samples, revealing a highly active distribution of the integrin family and its ligand receptor pairs in the longitudinal spatial sample (Fig. 7F). We also used CellChat to evaluate spatial cell–cell communication among the 12 segments in the longitudinal spatial sample and found that the VISFATIN signalling pathway is active in the caudal region (Fig. 7G), which is closely related to visfatin. Combined with the expression of *Apod*, the fatty acid metabolism and transport during sperm maturation deserve further research. In addition, the epididymal duct at the cauda region of the epididymis was surrounded by a ring of smooth muscle cells expressing the muscle cell-specific genes, myosin, heavy polypeptide 11 (*Myh11*), and transgelin (*Tagln*) (Fig. 7H and I, Fig. S8C and D). This was confirmed by FISH containing *Defb9* mRNA with MYH11, in the anterior caudal epididymis, there is only a thin layer of smooth muscle cells surrounding the epididymal epithelium, while in the posterior caudal epididymis, the smooth muscle cells become thicker (Fig. S8E, Fig. 7J), suggesting that this may be related to ejaculatory contraction. In summary, our data provided a full view at single-cell and visual-spatial level to study the cellular environment and gene expression profile of mouse testis and epididymis, as well as high region specificity of beta-defensin family genes.

## Discussion

Sperm development is a complex and long-term process, with stages from spermatogonia to fertile sperm required to undergo meiosis, sperm maturation, motility, and long-distance swimming for fertilisation [49, 50]. These processes are closely related to the cellular and molecular environments in which sperm live, making it important to have a comprehensive understanding of sperm development and its living environment. In this study, we integrated single-cell and spatial transcriptome sequencing techniques to delineate the distinct cell types within the testis and epididymis, particularly in the context of spermatogenesis and maturation. We found that epididymal epithelium cells displayed a region-specific gene expression patterns, especially in beta-defensin gene family. Further investigation revealed that mRNA of certain beta-defensin genes is abundant in principal cells and sperm head, which also showing a close connection to epididimosome. These results provide novel insights into sperm maturation.

We investigated the cellular components of mouse testes and profiled their gene expression characteristics, ranging from SPG in early sperm development to spermatozoa at later stages, as well as their communication with sertoli and leydig cells. In the epididymis, epithelial cells exhibit distinct regional specificity, stemming not only from their structural isolation but also from substantial variations in gene expression profiles. Additionally, we acquired a comprehensive gene expression landscape for different epididymal regions. In this study, we divided the epididymis into 12 distinct segments based on their structural barriers and gene expression characteristics. In addition to the top genes, we also investigated the entire gene expression profile in these 12 regions, which provided the basis for the subsequent study of the expression of specific genes in different regions of the epididymis at single-cell and spatial levels.

Studies have demonstrated the function of beta-defensin genes in epididymis [14, 16, 18, 23, 29, 51]. On one hand, *Spag11a* and *Defb15* in rat were reported to play roles in sperm motility acquisition and maintenance [14, 15]. On the other hand, deletion of a adjacent-subset of beta-defensin genes in the chromosome 8 would lead to disfunction of the acrosome reaction and intracellular calcium regulation in sperm [29]. Therefore, beta-defensins can be used as ideal markers for assessing sperm fertilization ability [52]. Beta-defensins are also reported to display a epididymal specificity in different regions [24, 53], suggesting their potential influence on sperm function through synergistic and sequential mechanisms [23]. These findings have brought important implications for future research. However, some of these studies obtained regional gene expression information by cutting the epididymal tissues into small pieces for bulk RNA sequencing. This leads to two limitations, one is that gene expression patterns in tissues are continuous, and piece-regional sequencing does not allow a clear assessment of gene expression initiation and decay information, especially in elongated tissues such as the epididymis. Another limitation is that bulk RNA sequencing of tissue pieces cannot recognise the subcluster of interest from mixed cell populations. Furthermore, single-cell data offer detailed gene expression profiles at high resolution but lack spatial location information. In our study, we performed single-cell and spatial transcriptomic sequencing synchronously, investigating cell types in testicular and epididymal tissues and their gene expression profiles at the single-cell level. We also used Cell2location analysis to map these cell types from scRNA-seq data on the spatial samples and obtained the spatial distribution of the epididymal epithelium, including six principal cell subsets. In addition, the complete crosscut and longitudinal spatial samples provided us with a continuous and visual-image spatial gene expression profile of the epididymis, including beta-defensins. For example, *Defb18* and *Defb15* are quite specific in different regions of caput epididymis; while the expression of *Defb30* originates at the rear region of caput epididymis and wear off at the corpus; in the caudal epididymis, *Defb2* and *Defb9* are activate in principal cells, which are surrounded by *Myh11* ^+^ and *Tagln* ^+^ smooth muscle cells. These data exhibit a continuous and direct cognition for us to understand the underlying mechanisms of the expression patterns and regulation of beta-defensin family genes.

As previously mentioned, beta-defensins exhibit regional specificity within the epididymis and play crucial roles in sperm maturation and acquisition of motility; however, little is known about the detailed mechanism of beta-defensins function. As we known, proteins and other components synthesiszed by epididymal epithelial cells are secreted into the epididymal fluid via the apical plasma. Simultaneously, the formed epididymosomes transport secretions to the sperm and directly participate in the regulation of sperm maturation [7, 47, 48]. Our data demonstrated that *Defb20* mRNA was located in caput principal cells and interestingly, *Defb20* mRNA was also found to be abundant in sperm head, and this distribution displayed a close connection to the epididymosome marker gene *Cd63*. Several studies have reported that while sperm do not transcribe new mRNA, they can utilize nuclear-encoded mRNA transcripts for protein translation through mitochondrial-type ribosomes [54, 55]. On the other hand, Li et al. found that most of cell-free seminal mRNA (cfs-mRNAs) existed in epididimosomes, which has the ability to deliver RNAs or proteins to sperm [56, 57]. Nonetheless, the ability of mRNAs in epididimosomes to translate proteins remains controversial because of the lack of powerful and direct evidence. For the mRNA of beta-defensins, it remains unverified whether these mRNA transported by epididymosomes possesses translational capacity and contributes to sperm maturation; nevertheless, it should be cautious to ignore the fact that there is abundant *Defb20* mRNA in sperm head within lumen of epididymis. Further investigation is required to elucidate the functioning of the beta-defensin gene family in sperm maturation and capacitation.

Overall, our study revealed complex gene expression and cellular composition in the testis and epididymis, especially, beta-defensin family genes exhibit regional specificity in the epididymis. In addition, we investigated other cellular components of the epididymis, such as fibroblasts and smooth muscle cells, and determined their distribution and gene expression levels. Thus, we obtained a panoramic landscape of the testis and epididymis to describe the cellular and molecular environment during sperm development, providing a basis for a better understanding of spermatogenesis and sperm maturation.

## Materials and methods

### Experimental animals

All animals used in this study were housed and maintained at a temperature of 23 °C and light (12 h light and 12 h dark) in a controlled room with ad libitum access to food and water. 6-week-old C57BL/6N mice were used to collect testicular and epididymal samples purchased from Vital River Laboratory Animal Technology Co., LTD.(Beijing, China). All animal experiments were approved by the Ethics Committee of Qingdao Agricultural University.

### Hematoxylin–eosin (H&E) staining

Mouse testicular and epididymal samples were collected, washed three times with phosphate-buffered saline (PBS; Sangon, Shanghai, China), fixed in paraformaldehyde (Servicebio, Wuhan, China), and embedded in paraffin (Sangon, Shanghai, China). The 5-μm sections were dewaxed and rehydrated and then stained with haematoxylin (Servicebio, Wuhan, China) solution for 3–5 min., Followed by staining with eosin (ServiceBio, Wuhan, China) for 3 min. The sections were dehydrated in 85% ethanol (Sangon, Shanghai, China) and 95% ethanol for 5 min. The sections were then dehydrated three times in 100% ethanol for 5 min each and sealed with neutral balsam (Sangon, Shanghai, China).

### Single-cell RNA-seq with the DNBelab C4 system

The entire epididymis from the four mice was cleaned and washed three times with PBS and then divided into three parts: the caput, corpus, and cauda. The samples were stored in MACS Tissue Storage Solution for 48 h (Miltenyi, Bergisch Gladbach, Germany). Before dissociation, the epididymal tissues were cut into small pieces and transferred to 0.2% collagenase IV (1 mg/ml; Sangon, Shanghai, China) and DNase I (5 U/ml; Sangon, Shanghai, China) digestion solutions, followed by incubation at 37 °C for 15 min. After digestion and mechanical striking of single cells, the cell suspension was filtered through a cell strainer (Solarbio, Beijing, China) and converted into barcoded scRNA-seq libraries, according to the manufacturer’s protocol. The libraries were sequenced using the DNBSEQ-T7 (Mgi Tech, Shenzhen, China).

### Visium spatial transcriptome sequencing

The fixed samples from mouse testicular and epididymal samples were embedded in paraffin and sliced into 5 µm sections. Following the FFPE Visium workflow, the FFPE sections were placed on Superfrost™ Plus Microscope Slides (Fisherbrand™) for deparaffinisation. Experimental slides of the testicular and epididymal samples were fixed, stained with haematoxylin and eosin and scanned to obtain full-view images of the tissues. The sections were destained with haematoxylin and de-crosslinked, and the analytes were transferred onto Visium CytAssist Spatial Gene Expression slides. Sequence libraries were processed using the FFPE Visium workflow. Libraries were sequenced using the Illumina NovaSeq 6000 (ANOROAD, Beijing, China) platform.

### Data processing and analysis

Gene expression matrices of the four scRNA-seq samples were loaded into R (V4.2.2) and merged for the following analysis by the package “Seurat” v4.3.0 (https://satijalab.org) [58]. The merged data was filtered to include cells with too few genes and not more than 6500 (300 < nFeature_RNA < 6500) and less than 20% of mitochondrial transcripts (percent.mt < 20). Then the filtered data were Log-normalized and scaled, and then visualized through dimensionality reduction by the RunUMAP function. For reanalyzing testicular population or epididymal population, cell clusters from the testis or different segments of the epididymis were extracted from the merged dataset by the SubsetData function. The extracted cluster was divided into several subclusters according to their specific gene expression patterns. We used VlnPlot, FeaturePlot and DoHeatmap fuction of Seurat to find out the gene expression profiles in each subcluster. For the visium spatial samples of epididymis, the gene expression data and image information were loaded into Seurat and then normalized by SCTransform function to view spatial expression information of certain genes. Followed by PCA analysis and RunUMAP processing step, the cluster of spots on spatial samples can be identified and processed further.

### Transmission electron microscopy (TEM)

The segmented epididymal samples (caput, corpus, and cauda) were dissected and washed three times with PBS and fixed in 2.5% glutaraldehyde (0.2 M PBS, pH = 7.2; Sangon, Shanghai, China) overnight at 4 °C. The epididymal samples underwent dehydration using a series of alcohol concentrations: 30%, 50%, 70%, 80%, 95%, 100%, and two rounds of 100% acetone, each lasting 20 min for alcohol and 15 min for acetone. The treated samples were embedded in epoxypropane resin, sliced into 50 nm sections using an EM UC7 ultramicrotome (Leica, Wetzlar, Germany), stained with lead citrate and uranium, and observed using an HT7700 TEM (Hitachi, Tokyo, Japan).

### Immunofluorescence

Mouse testicular and epididymal samples were collected, washed three times with PBS, fixed in 4% paraformaldehyde at 4 °C overnight, embedded in paraffin, and sliced into 5 μm sections (Leica, Wetzlar, Germany). After dewaxing and rehydration, the prepared sections were repaired in citric acid antigen retrieval solution (Servicebio, Wuhan, China) at 96 °C for 10 min and cooled naturally to room temperature. Then, the sections were washed with prewarmed Tris Buffered Saline (TBS;Servicebio, Wuhan, China), blocked with QuickBlock™ Blocking Buffer (Beyotime, Shanghai, China) for Immunol Staining for 10 min, and incubated with the primary antibody overnight at 4 °C. The primary antibodies used were a Recombinant Anti-CD63 antibody (dilution 1:100, #Ab217345; Abcam, Cambridge, UK) and SMMHC/MYH11 Rabbit pAb (dilution 1:100, #A10827; ABclonal, Wuhan, China). After incubation, the sections were washed three times with TBST (Servicebio, Wuhan, China) and then incubated with secondary antibody for 30 min at 37 °C. Goat Anti-Rabbit IgG (dilution 1:50, #AS060; ABclonal, Wuhan, China) was used as the secondary antibody. Cell nuclei were labelled with Antifade Mounting Medium containing DAPI (dilution 1:1000; Beyotime, Shanghai, China).

### *SweAMI probe *in situ* hybridisation*

The Saiweier signal Amplification Multiplex Isothermal (SweAMI) probes were designed and synthesized by Servicebio (Wuhan, China). The mouse epididymal samples were collected and fixed in in-situ hybridization fixative for more that 12 h, and then embedded in paraffin and sliced into 5 μm sections. The sections were baked in an oven at 62 °C for 2 h and placed in xylene (Sangon, Shanghai, China) for 30 min for dewaxing. The sections were then rehydrated and repaired in a citric acid antigen retrieval solution (Servicebio, Wuhan, China) at 96 °C for 10 min and cooled naturally to room temperature. Proteinase K (20 µg/ml; Servicebio, Wuhan, China) was added dropwise to the sections for a 10-min digestion at 37 °C. The sections were subjected to prehybridisation and hybridisation in a solution containing the probe dropwise, washed in 2 × SSC (Servicebio, Wuhan, China) for 10 min at 37 ℃ and in 1 × SSC twice for 5 min each at 37 ℃, and washed in 0.5 × SSC for 10 min at room temperature. The RNA probes and sequences used include *Defb9* (5'UTR:CAC CGT TCC ATT TCT GAT ACA CCG ATT GAG AAC TGA AAC AAT AAA AAT AAC AGT ACC CT), *Defb19* (5'UTR:GAC CAG TTC CGT TGC CAC AAG TAT GTT GAA GGA TAG GAT TTT TGC CAG ACG TAG GCC TGT TCA CTC TTT TTG CAG), *Defb20* (5'UTR:CAC CAT CTG CAA GTG CCA CAA ACA GTT CTA CGT TGC TAA AAC ATC TTT TGG GCT CCA CTA ATC TGC ATT TCT TCC TAC AG), and *Defb30* (5'UTR:AAG AGC ACG AGG GTC AAC TGT AGG CAC TGG TGG AAC ATA GGA GAG CAA GAG CAT GTG TCG TAA ATC CGT TTT ATG TTC CTG CAA ATG CCT TTT AGT TTC CAA CGA CTG AAT GCC ACA GAA AAT GT). For double staining with antibodies, the sections were blocked with QuickBlock™ Blocking Buffer for Immunol Staining (Beyotime, Shanghai, China) for 10 min and incubated with the primary antibody overnight at 4 °C. After washing thrice with TBST, the sections were incubated with the secondary antibody and treated with an Antifade Mounting Medium containing DAPI (Beyotime, Shanghai, China).

### Supplementary Information

Supplementary Material 1. Fig. S1 Proportion of different cell types and detail subsets of all cells in testicular and epididymal tissues. Related to Figure 1.A) Proportion of 10 major cell types showing in bar plots from different samples. B) Violin plots of associated gene expression across 9 subsets of testicular cells. C) Feature plots of representative marker genes across testicular cells.

Supplementary Material 2. Fig. S2 Beta-defensins are active in testis. Related to Figure 2. A) Feature plots of *Defb33* gene expression in testicular single-cell sequencing data. B-D) Spatial gene expression features of *Defb33*, *Defb19*, *Defb36 *in crosscut spatial sample, respectively. E) Representative FISH staining of *Defb19* mRNA in testis. Scale bar: 50 μm.

Supplementary Material 3. Fig. S3 Mapping spatial data with scRNA-Seq cell type annotations by using Cell2location in the crosscut spatial sample. Related to Figure 3.

Supplementary Material 4. Fig. S4 Spatial gene expression features of rest member of beta-defensin gene family in the crosscut spatial sample. Related to Figure 4.

Supplementary Material 5. Fig. S5Spatial gene expression features of rest member of beta-defensin gene family in the longitudinal spatial sample. Related to Figure 5.

Supplementary Material 6. Fig. S6 Mapping spatial data with scRNA-Seq cell type annotations by using Cell2location in the longitudinal spatial sample. Related to Figure 5. A) Mapping spatial data with scRNA-Seq cell type annotations of epididymal epithelium by using Cell2location. B) Mapping spatial data with scRNA-Seq cell type annotations of stromal cells by using Cell2location. C) Mapping spatial data with scRNA-Seq cell type annotations of all cells by using Cell2location.

Supplementary Material 7. Fig.S7 Beta-defensin gene showed region specificity in the mouse epididymal tissues. Related to Figure 6. A) Spatial gene expression features of lipocalin 8* (Lcn8)*, lipocalin 9* (Lcn9)*, *Cst12,* and *Cd63* in the longitudinal spatial sample. B) Representative IF staining of CD63 and FISH staining of *Defb30* mRNA in the corpus region of epididymis. Scale bar: 50 μm. C) Representative IF staining of CD63 and FISH staining of *Defb9* mRNA in the cauda region of epididymis. Scale bar: 50 μm. D) Representative IF staining of DEFB121 in the caput epididymis. Scale bar: 300 μm. E) Enlarged view of Fig. S7D (white frame), showing DEFB121 is positive in principal cells. Scale bar: 25 μm. F) Negative control of DEFB121 IF staining (without primary antibody). Scale bar: 25 μm. G) Representative IF staining of DEFB121 in the posterior region of caput. Scale bar: 50 μm.

Supplementary Material 8. Fig. S8 Gene expression profile of stromal cells in mouse epididymis. Related to Figure 7. A) Spatial gene expression feature of *Col6a5* in longitudinal spatial sample. B) Spatial gene expression feature of *Apod* in crosscut and longitudinal spatial samples. C) Spatial gene expression feature of *Tagln* in crosscut and longitudinal spatial samples. D) Spatial gene expression feature of *Myh11* in longitudinal spatial sample. E) Representative IF staining of MYH11 and FISH staining of *Defb9* mRNA in the front-end region of caudal epididymis.

Supplementary Material 9. 

Supplementary Material 10. 

Supplementary Material 11. 

Supplementary Material 12. 

## Data Availability

The raw sequence data reported in this paper have been deposited in the Genome Sequence Archive [59] at the National Genomics Data Center [60], China National Center for Bioinformation/Beijing Institute of Genomics, Chinese Academy of Sciences (GSA: CRA012263), and are publicly accessible at https://ngdc.cncb.ac.cn/gsa. Additional information related to the data used in this study is available from the lead contact upon request.

## References

[1] Grunewald S, Paasch U, Glander HJ, Anderegg U (2005). Mature human spermatozoa do not transcribe novel RNA. Andrologia.

[2] Dacheux JL, Dacheux F (2014). New insights into epididymal function in relation to sperm maturation. Reproduction.

[3] Cooper TG (2015). Epididymal research: more warp than weft?. Asian J Androl.

[4] Gervasi MG, Visconti PE (2017). Molecular changes and signaling events occurring in spermatozoa during epididymal maturation. Andrology.

[5] Breton S, Ruan YC, Park YJ, Kim B (2016). Regulation of epithelial function, differentiation, and remodeling in the epididymis. Asian J Androl.

[6] Sullivan R, Legare C, Lamontagne-Proulx J, Breton S, Soulet D (2019). Revisiting structure/functions of the human epididymis. Andrology.

[7] Barrachina F, Battistone MA, Castillo J, Mallofre C, Jodar M, Breton S, Oliva R (2022). Sperm acquire epididymis-derived proteins through epididymosomes. Hum Reprod.

[8] Cyr DG, Robaire B, Hermo L (1995). Structure and turnover of junctional complexes between principal cells of the rat epididymis. Microsc Res Tech.

[9] Leung GP, Cheung KH, Leung CT, Tsang MW, Wong PY (2004). Regulation of epididymal principal cell functions by basal cells: role of transient receptor potential (Trp) proteins and cyclooxygenase-1 (COX-1). Mol Cell Endocrinol.

[10] Wang H, Kumar TR (2012). Segment- and cell-specific expression of D-type cyclins in the postnatal mouse epididymis. Gene Expr Patterns.

[11] Breton S, Nair AV, Battistone MA (2019). Epithelial dynamics in the epididymis: role in the maturation, protection, and storage of spermatozoa. Andrology.

[12] Ren D, Navarro B, Perez G, Jackson AC, Hsu S, Shi Q, Tilly JL, Clapham DE (2001). A sperm ion channel required for sperm motility and male fertility. Nature.

[13] Dacheux JL, Belleannee C, Guyonnet B, Labas V, Teixeira-Gomes AP, Ecroyd H, Druart X, Gatti JL, Dacheux F (2012). The contribution of proteomics to understanding epididymal maturation of mammalian spermatozoa. Syst Biol Reprod Med.

[14] Zhao Y, Diao H, Ni Z, Hu S, Yu H, Zhang Y (2011). The epididymis-specific antimicrobial peptide beta-defensin 15 is required for sperm motility and male fertility in the rat (Rattus norvegicus). Cell Mol Life Sci.

[15] Zhou CX, Zhang YL, Xiao L, Zheng M, Leung KM, Chan MY, Lo PS, Tsang LL, Wong HY, Ho LS (2004). An epididymis-specific beta-defensin is important for the initiation of sperm maturation. Nat Cell Biol.

[16] Fernandez-Fuertes B, Narciandi F, O'Farrelly C, Kelly AK, Fair S, Meade KG, Lonergan P (2016). Cauda Epididymis-Specific Beta-Defensin 126 Promotes Sperm Motility but Not Fertilizing Ability in Cattle. Biol Reprod.

[17] Diao R, Fok KL, Chen H, Yu MK, Duan Y, Chung CM, Li Z, Wu H, Li Z, Zhang H (2014). Deficient human beta-defensin 1 underlies male infertility associated with poor sperm motility and genital tract infection. Sci Transl Med..

[18] Zupin L, Polesello V, Martinelli M, Luppi S, Giolo E, Zito G, Romano F, Segat L, Crovella S, Ricci G (2019). Human beta-defensin 1 in follicular fluid and semen: impact on fertility. J Assist Reprod Genet.

[19] Li P, Chan HC, He B, So SC, Chung YW, Shang Q, Zhang YD, Zhang YL (2001). An antimicrobial peptide gene found in the male reproductive system of rats. Science.

[20] Wu P, Liu TL, Li LL, Liu ZP, Tian LH, Hou ZJ (2021). Declined expressing mRNA of beta-defensin 108 from epididymis is associated with decreased sperm motility in blue fox (Vulpes lagopus). BMC Vet Res.

[21] Ganz T (2003). Defensins: antimicrobial peptides of innate immunity. Nat Rev Immunol.

[22] Oppenheim JJ, Biragyn A, Kwak LW, Yang D (2003). Roles of antimicrobial peptides such as defensins in innate and adaptive immunity. Ann Rheum Dis..

[23] Ribeiro CM, Silva EJ, Hinton BT, Avellar MC (2016). beta-defensins and the epididymis: contrasting influences of prenatal, postnatal, and adult scenarios. Asian J Androl.

[24] Dorin JR, Barratt CL (2014). Importance of beta-defensins in sperm function. Mol Hum Reprod.

[25] Hall SH, Yenugu S, Radhakrishnan Y, Avellar MC, Petrusz P, French FS (2007). Characterization and functions of beta defensins in the epididymis. Asian J Androl.

[26] Yudin AI, Generao SE, Tollner TL, Treece CA, Overstreet JW, Cherr GN (2005). Beta-defensin 126 on the cell surface protects sperm from immunorecognition and binding of anti-sperm antibodies. Biol Reprod.

[27] Johnston DS, Jelinsky SA, Bang HJ, DiCandeloro P, Wilson E, Kopf GS, Turner TT (2005). The mouse epididymal transcriptome: transcriptional profiling of segmental gene expression in the epididymis. Biol Reprod.

[28] Diao H, Yu HG, Sun F, Zhang YL, Tanphaichitr N (2011). Rat recombinant beta-defensin 22 is a heparin-binding protein with antimicrobial activity. Asian J Androl.

[29] Zhou YS, Webb S, Lettice L, Tardif S, Kilanowski F, Tyrrell C, Macpherson H, Semple F, Tennant P, Baker T (2013). Partial deletion of chromosome 8 beta-defensin cluster confers sperm dysfunction and infertility in male mice. PLoS Genet.

[30] Zaballos A, Villares R, Albar JP, Martinez AC, Marquez G (2004). Identification on mouse chromosome 8 of new beta-defensin genes with regionally specific expression in the male reproductive organ. J Biol Chem.

[31] Belleannee C, Labas V, Teixeira-Gomes AP, Gatti JL, Dacheux JL, Dacheux F (2011). Identification of luminal and secreted proteins in bull epididymis. J Proteomics.

[32] Xie SW, Li GT, Qu LJ, Cao Y, Wang Q, Zhou JY, Zhong RH, Guo XJ, Zhu Y (2016). Identification of New Epididymal Luminal Fluid Proteins Involved in Sperm Maturation in Infertile Rats Treated by Dutasteride Using iTRAQ. Molecules..

[33] Sharma U, Conine CC, Shea JM, Boskovic A, Derr AG, Bing XY, Belleannee C, Kucukural A, Serra RW, Sun F (2016). Biogenesis and function of tRNA fragments during sperm maturation and fertilization in mammals. Science.

[34] Hutcheon K, McLaughlin EA, Stanger SJ, Bernstein IR, Dun MD, Eamens AL, Nixon B (2017). Analysis of the small non-protein-coding RNA profile of mouse spermatozoa reveals specific enrichment of piRNAs within mature spermatozoa. RNA Biol.

[35] Rinaldi VD, Donnard E, Gellatly K, Rasmussen M, Kucukural A, Yukselen O, Garber M, Sharma U, Rando OJ (2020). An atlas of cell types in the mouse epididymis and vas deferens. Elife..

[36] Shi J, Fok KL, Dai P, Qiao F, Zhang M, Liu H, Sang M, Ye M, Liu Y, Zhou Y (2021). Spatio-temporal landscape of mouse epididymal cells and specific mitochondria-rich segments defined by large-scale single-cell RNA-seq. Cell Discov.

[37] Guo J, Grow EJ, Mlcochova H, Maher GJ, Lindskog C, Nie X, Guo Y, Takei Y, Yun J, Cai L (2018). The adult human testis transcriptional cell atlas. Cell Res.

[38] Sohni A, Tan K, Song HW, Burow D, de Rooij DG, Laurent L, Hsieh TC, Rabah R, Hammoud SS, Vicini E, Wilkinson MF (2019). The Neonatal and Adult Human Testis Defined at the Single-Cell Level. Cell Rep.

[39] Lukassen S, Bosch E, Ekici AB, Winterpacht A (2018). Single-cell RNA sequencing of adult mouse testes. Sci Data.

[40] Rahrmann EP, Shorthouse D, Jassim A, Hu LP, Ortiz M, Mahler-Araujo B, Vogel P, Paez-Ribes M, Fatemi A, Hannon GJ (2022). The NALCN channel regulates metastasis and nonmalignant cell dissemination. Nat Genet.

[41] Hao Y, Hao S, Andersen-Nissen E, Mauck WM, Zheng S, Butler A, Lee MJ, Wilk AJ, Darby C, Zager M (2021). Integrated analysis of multimodal single-cell data. Cell.

[42] Weiser D, Mietens A, Stadler B, Jezek D, Schuler G, Middendorff R (2020). Contractions transport exfoliated epithelial cells through the neonatal epididymis. Reproduction.

[43] Kleshchevnikov V, Shmatko A, Dann E, Aivazidis A, King HW, Li T, Elmentaite R, Lomakin A, Kedlian V, Gayoso A (2022). Cell 2location maps fine-grained cell types in spatial transcriptomics. Nat Biotechnol.

[44] Li B, Zhang W, Guo C, Xu H, Li L, Fang M, Hu Y, Zhang X, Yao X, Tang M (2022). Benchmarking spatial and single-cell transcriptomics integration methods for transcript distribution prediction and cell type deconvolution. Nat Methods.

[45] Pujianto DA, Muliawati D, Rizki MD, Parisudha A, Hardiyanto L (2020). Mouse defensin beta 20 (Defb20) is expressed specifically in the caput region of the epididymis and regulated by androgen and testicular factors. Reprod Biol.

[46] Aram R, Chan PTK, Cyr DG (2020). Beta-defensin126 is correlated with sperm motility in fertile and infertile mendagger. Biol Reprod.

[47] D'Amours O, Frenette G, Bordeleau LJ, Allard N, Leclerc P, Blondin P, Sullivan R (2012). Epididymosomes transfer epididymal sperm binding protein 1 (ELSPBP1) to dead spermatozoa during epididymal transit in bovine. Biol Reprod.

[48] Frenette G, Lessard C, Sullivan R (2002). Selected proteins of "prostasome-like particles" from epididymal cauda fluid are transferred to epididymal caput spermatozoa in bull. Biol Reprod.

[49] Neto FT, Bach PV, Najari BB, Li PS, Goldstein M (2016). Spermatogenesis in humans and its affecting factors. Semin Cell Dev Biol.

[50] Staub C, Johnson L (2018). Review: Spermatogenesis in the bull. Animal.

[51] Zhang C, Zhou Y, Xie S, Yin Q, Tang C, Ni Z, Fei J, Zhang Y (2018). CRISPR/Cas9-mediated genome editing reveals the synergistic effects of beta-defensin family members on sperm maturation in rat epididymis. FASEB J.

[52] Solanki S, Kumar V, Kashyap P, Kumar R, De S, Datta TK (2023). Beta-defensins as marker for male fertility: a comprehensive reviewdagger. Biol Reprod.

[53] Johnston DS, Turner TT, Finger JN, Owtscharuk TL, Kopf GS, Jelinsky SA (2007). Identification of epididymis-specific transcripts in the mouse and rat by transcriptional profiling. Asian J Androl.

[54] Gur Y, Breitbart H (2006). Mammalian sperm translate nuclear-encoded proteins by mitochondrial-type ribosomes. Genes Dev.

[55] Gur Y, Breitbart H (2008). Protein synthesis in sperm: dialog between mitochondria and cytoplasm. Mol Cell Endocrinol.

[56] Li H, Huang S, Guo C, Guan H, Xiong C (2012). Cell-free seminal mRNA and microRNA exist in different forms. PLoS ONE.

[57] Wang L, Lv J, Guo C, Li H, Xiong C (2014). Recovery of cell-free mRNA and microRNA from human semen based on their physical nature. Biotechnol Appl Biochem.

[58] Butler A, Hoffman P, Smibert P, Papalexi E, Satija R (2018). Integrating single-cell transcriptomic data across different conditions, technologies, and species. Nat Biotechnol.

[59] Chen T, Chen X, Zhang S, Zhu J, Tang B, Wang A, Dong L, Zhang Z, Yu C, Sun Y (2021). The Genome Sequence Archive Family: Toward Explosive Data Growth and Diverse Data Types. Genomics Proteomics Bioinformatics.

[60] Members CN, Partners (2023). Database Resources of the National Genomics Data Center, China National Center for Bioinformation in 2023. Nucleic Acids Res.

