# Supplementary figures and images for: Single-cell and spatial transcriptomic investigation reveals the spatiotemporal specificity of the beta-defensin gene family during mouse sperm maturation
Source: Cell Commun Signal. 2024 May 14;22:267. doi: 10.1186/s12964-024-01637-3 (PMC11092205; doi:10.1186/s12964-024-01637-3)

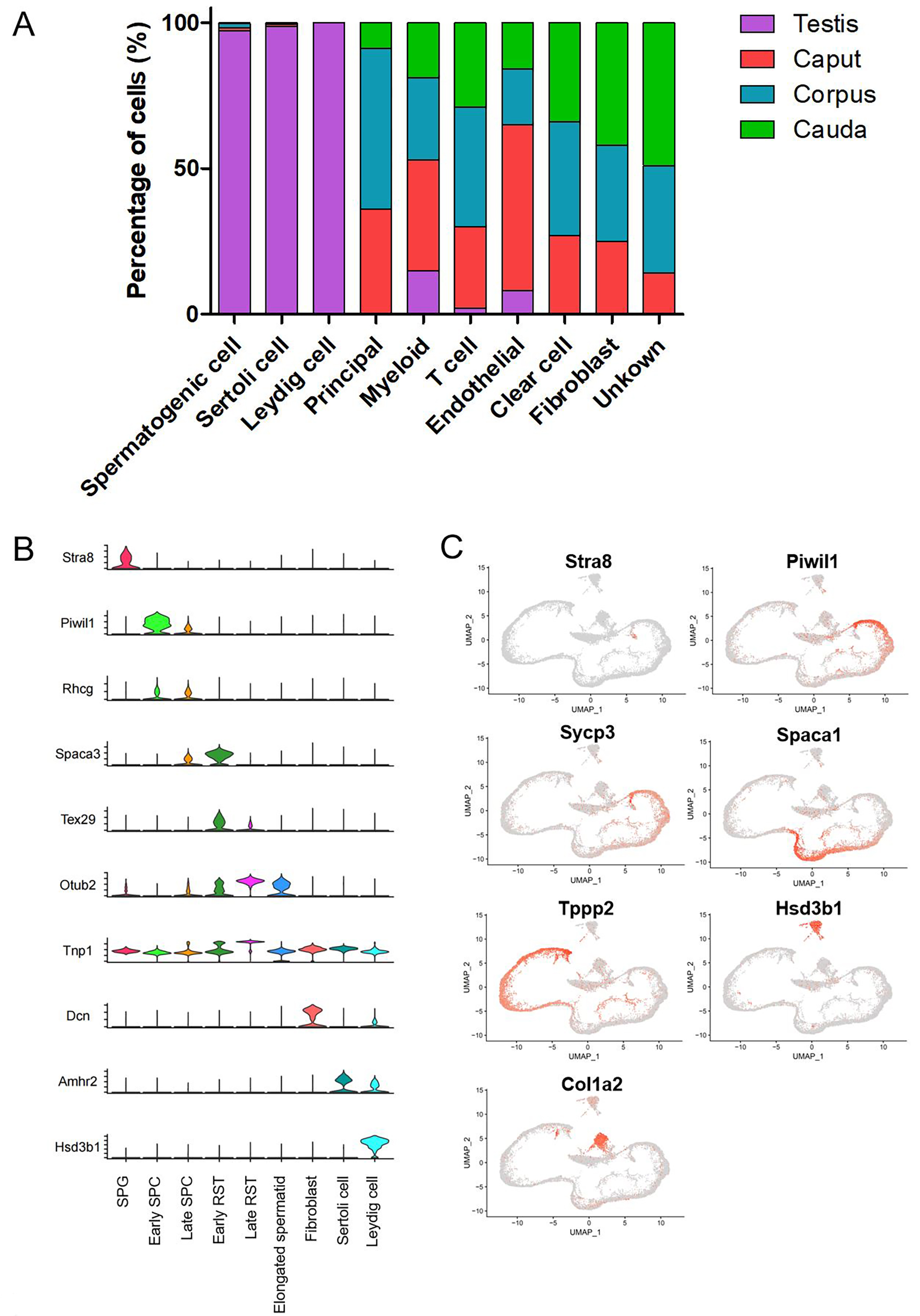

Supplement: Supplementary file 1 — Supplementary Material 1. Fig. S1 Proportion of different cell types and detail subsets of all cells in testicular and epididymal tissues. Related to Figure 1.A) Proportion of 10 major cell types showing in bar plots from different samples. B) Violin plots of associated gene expression across 9 subsets of testicular cells. C) Feature plots of representative marker genes across testicular cells. [file 12964_2024_1637_MOESM1_ESM.jpg]

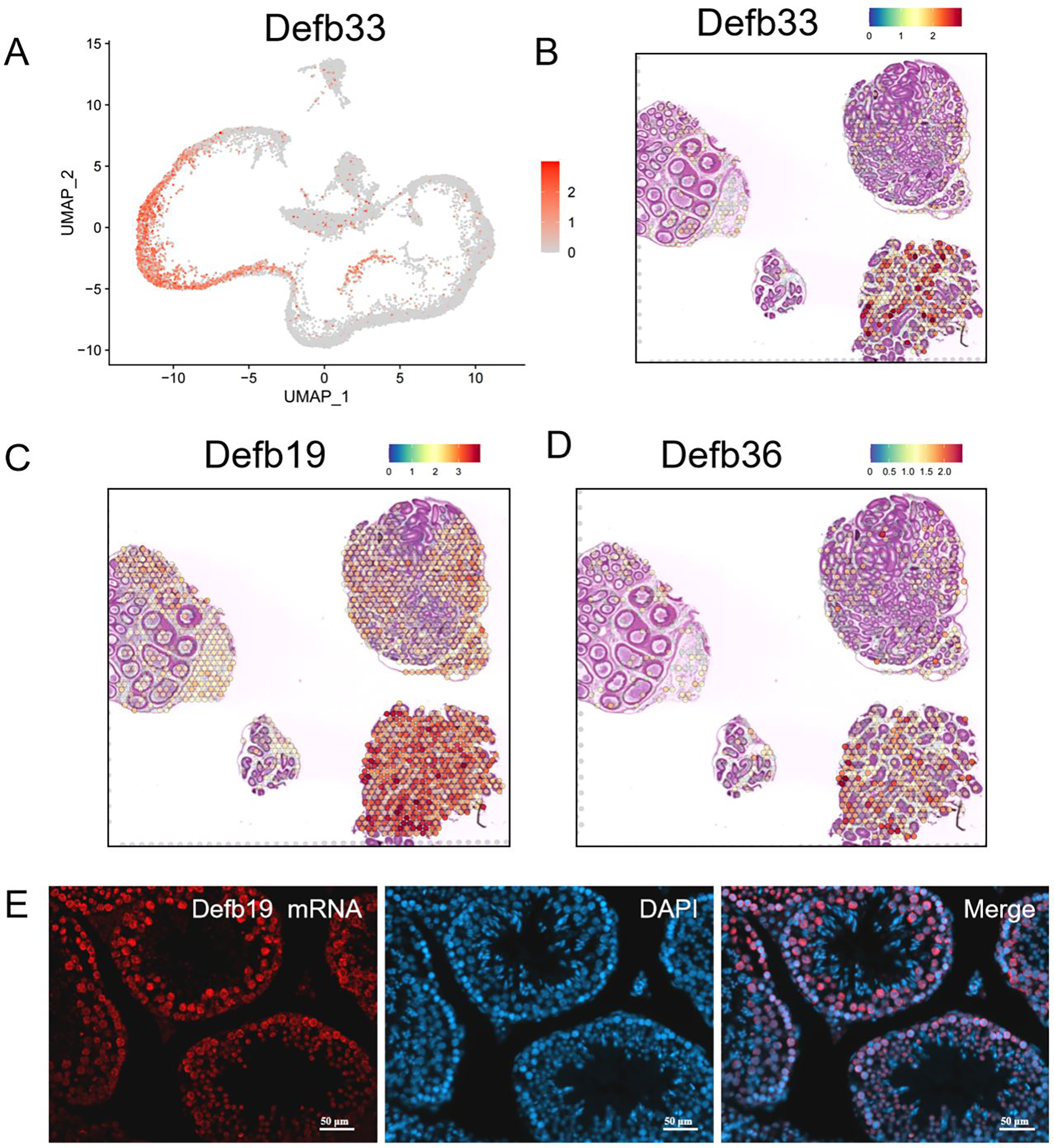

Supplement: Supplementary file 2 — Supplementary Material 2. Fig. S2 Beta-defensins are active in testis. Related to Figure 2. A) Feature plots of Defb33 gene expression in testicular single-cell sequencing data. B-D) Spatial gene expression features of Defb33, Defb19, Defb36 in crosscut spatial sample, respectively. E) Representative FISH staining of Defb19 mRNA in testis. Scale bar: 50 μm. [file 12964_2024_1637_MOESM2_ESM.jpg]

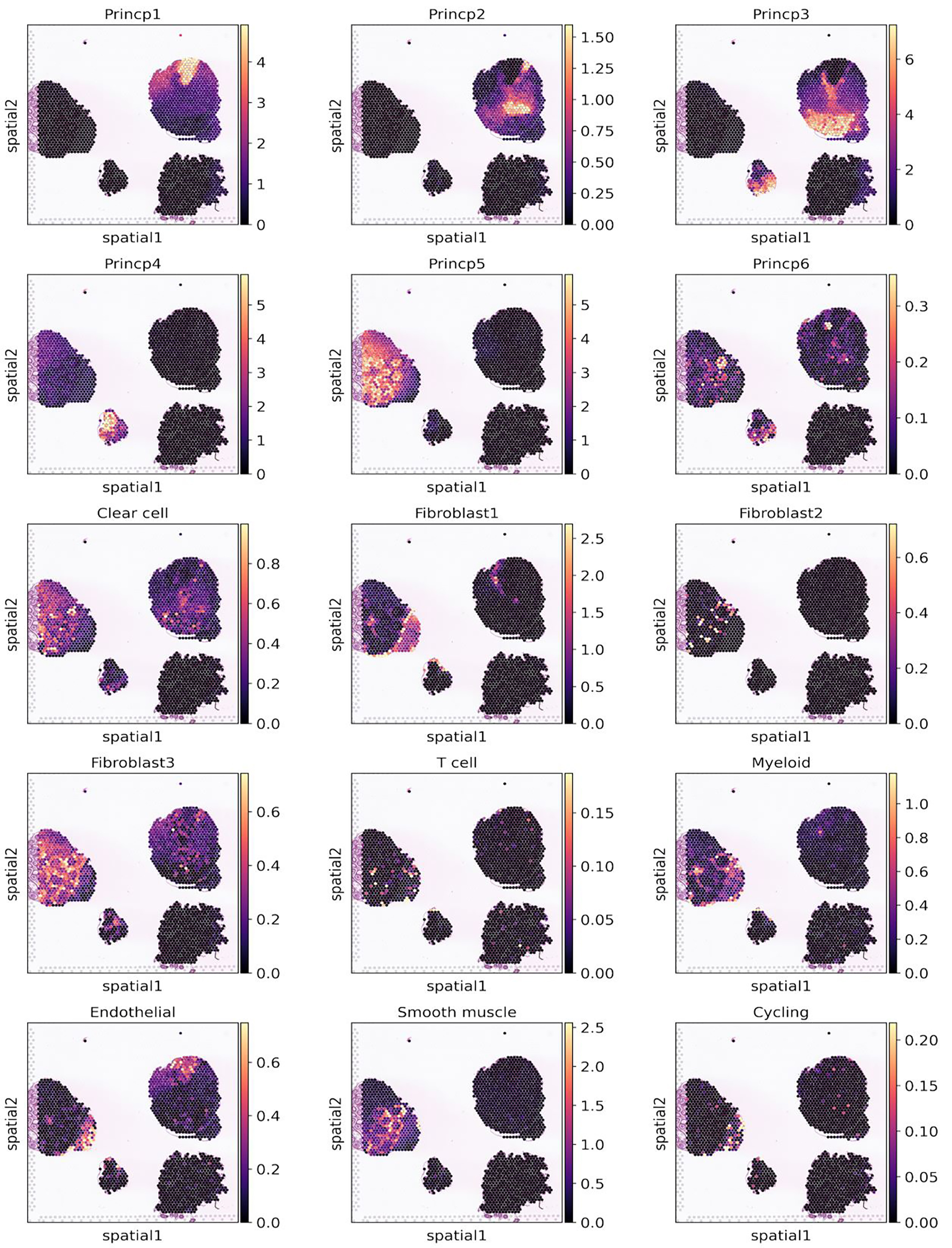

Supplement: Supplementary file 3 — Supplementary Material 3. Fig. S3 Mapping spatial data with scRNA-Seq cell type annotations by using Cell2location in the crosscut spatial sample. Related to Figure 3. [file 12964_2024_1637_MOESM3_ESM.jpg]

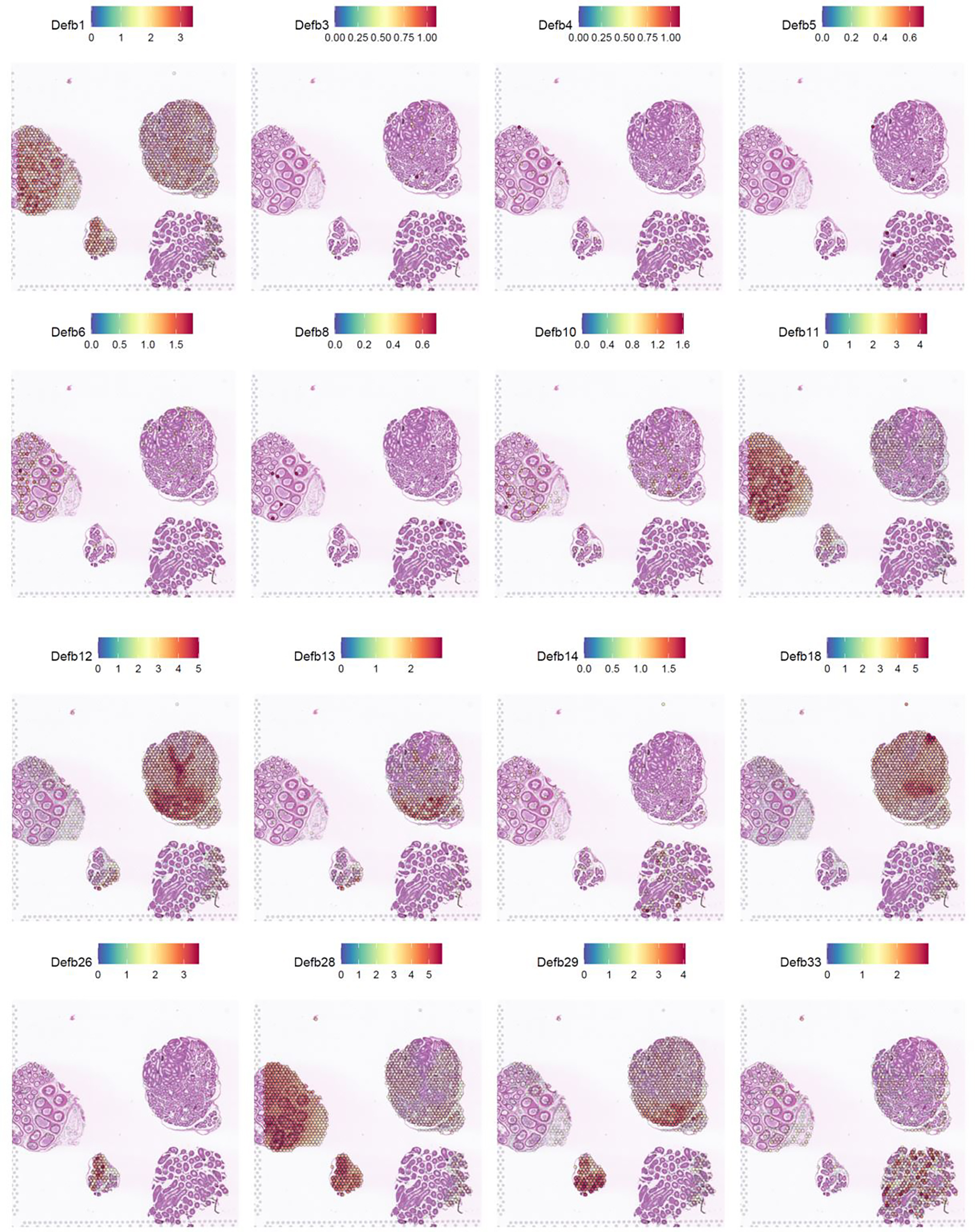

Supplement: Supplementary file 4 — Supplementary Material 4. Fig. S4 Spatial gene expression features of rest member of beta-defensin gene family in the crosscut spatial sample. Related to Figure 4. [file 12964_2024_1637_MOESM4_ESM.zip › Figure S4-1.jpg]

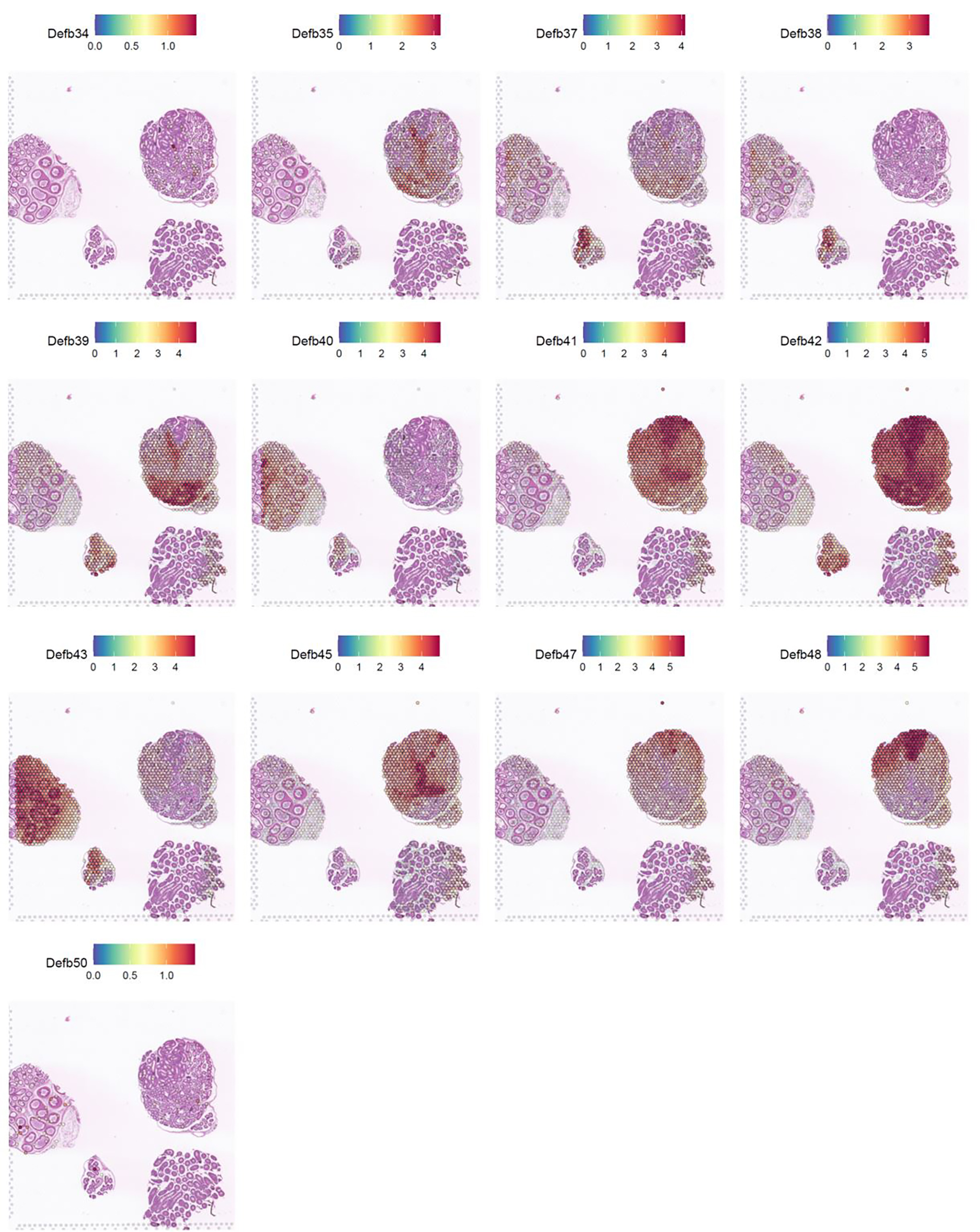

Supplement: Supplementary file 4 — Supplementary Material 4. Fig. S4 Spatial gene expression features of rest member of beta-defensin gene family in the crosscut spatial sample. Related to Figure 4. [file 12964_2024_1637_MOESM4_ESM.zip › Figure S4-2.jpg]

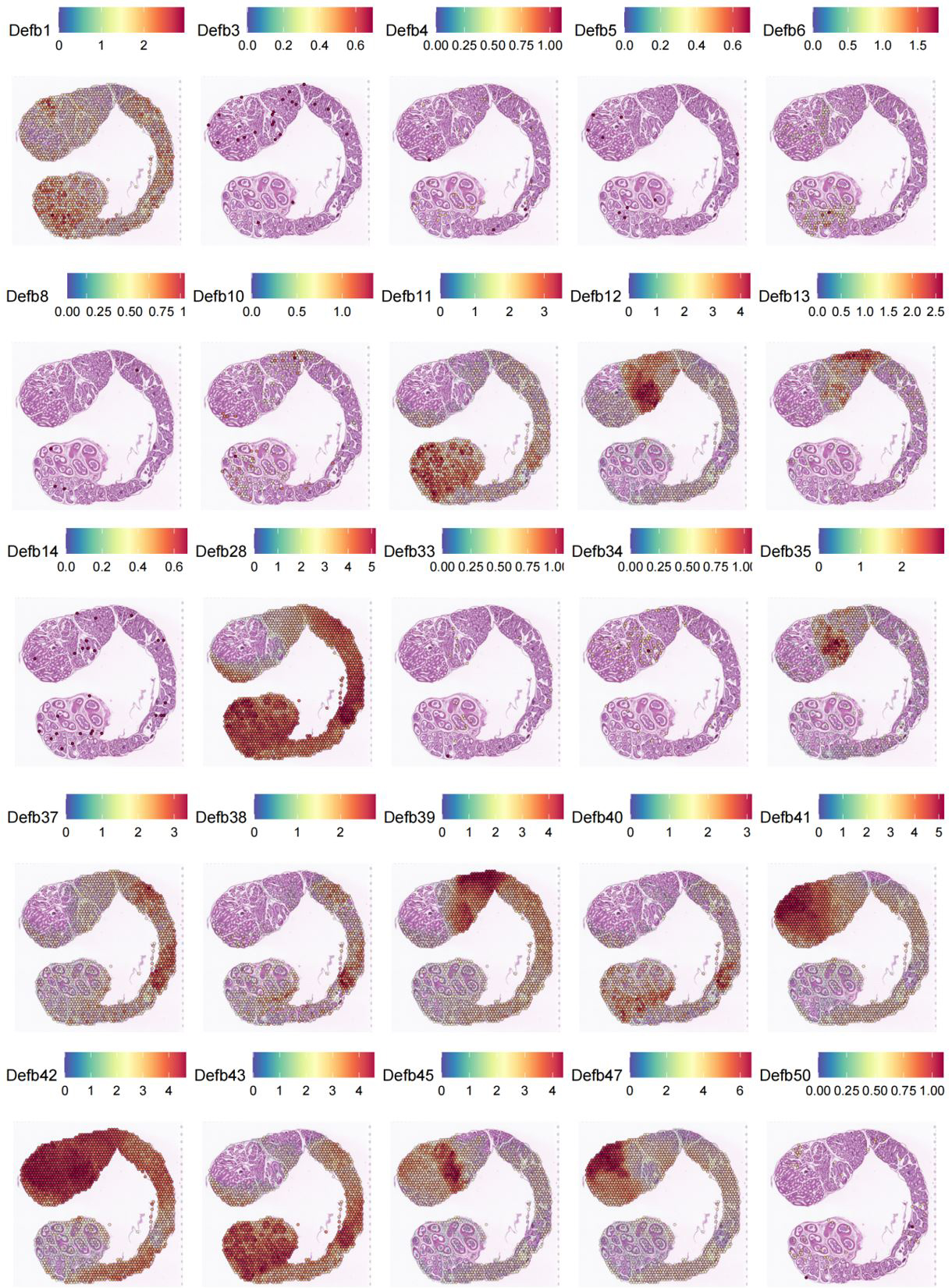

Supplement: Supplementary file 5 — Supplementary Material 5. Fig. S5Spatial gene expression features of rest member of beta-defensin gene family in the longitudinal spatial sample. Related to Figure 5. [file 12964_2024_1637_MOESM5_ESM.jpg]

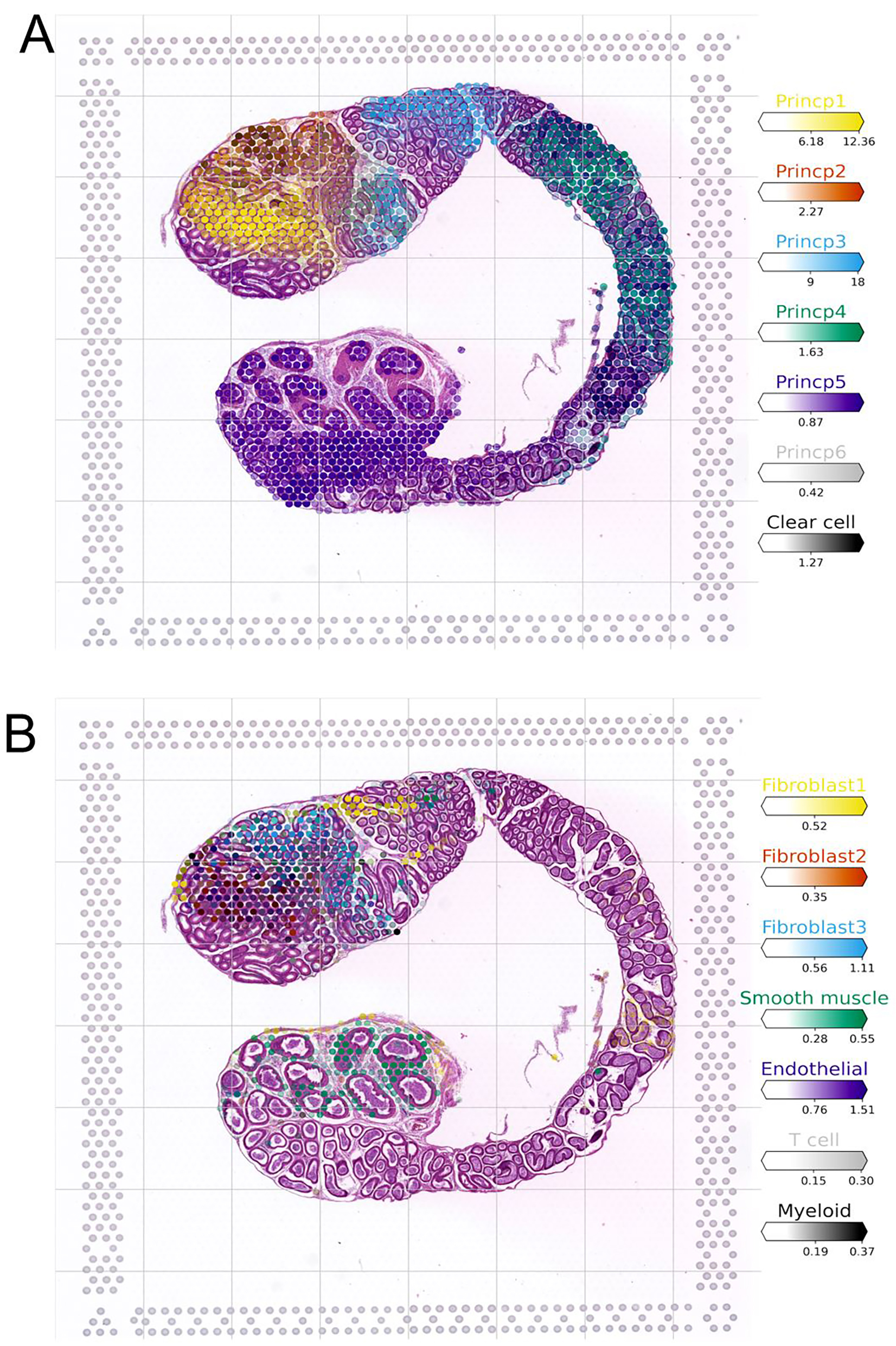

Supplement: Supplementary file 6 — Supplementary Material 6. Fig. S6 Mapping spatial data with scRNA-Seq cell type annotations by using Cell2location in the longitudinal spatial sample. Related to Figure 5. A) Mapping spatial data with scRNA-Seq cell type annotations of epididymal epithelium by using Cell2location. B) Mapping spatial data with scRNA-Seq cell type annotations of stromal cells by using Cell2location. C) Mapping spatial data with scRNA-Seq cell type annotations of all cells by using Cell2location. [file 12964_2024_1637_MOESM6_ESM.zip › Figure S6-1.jpg]

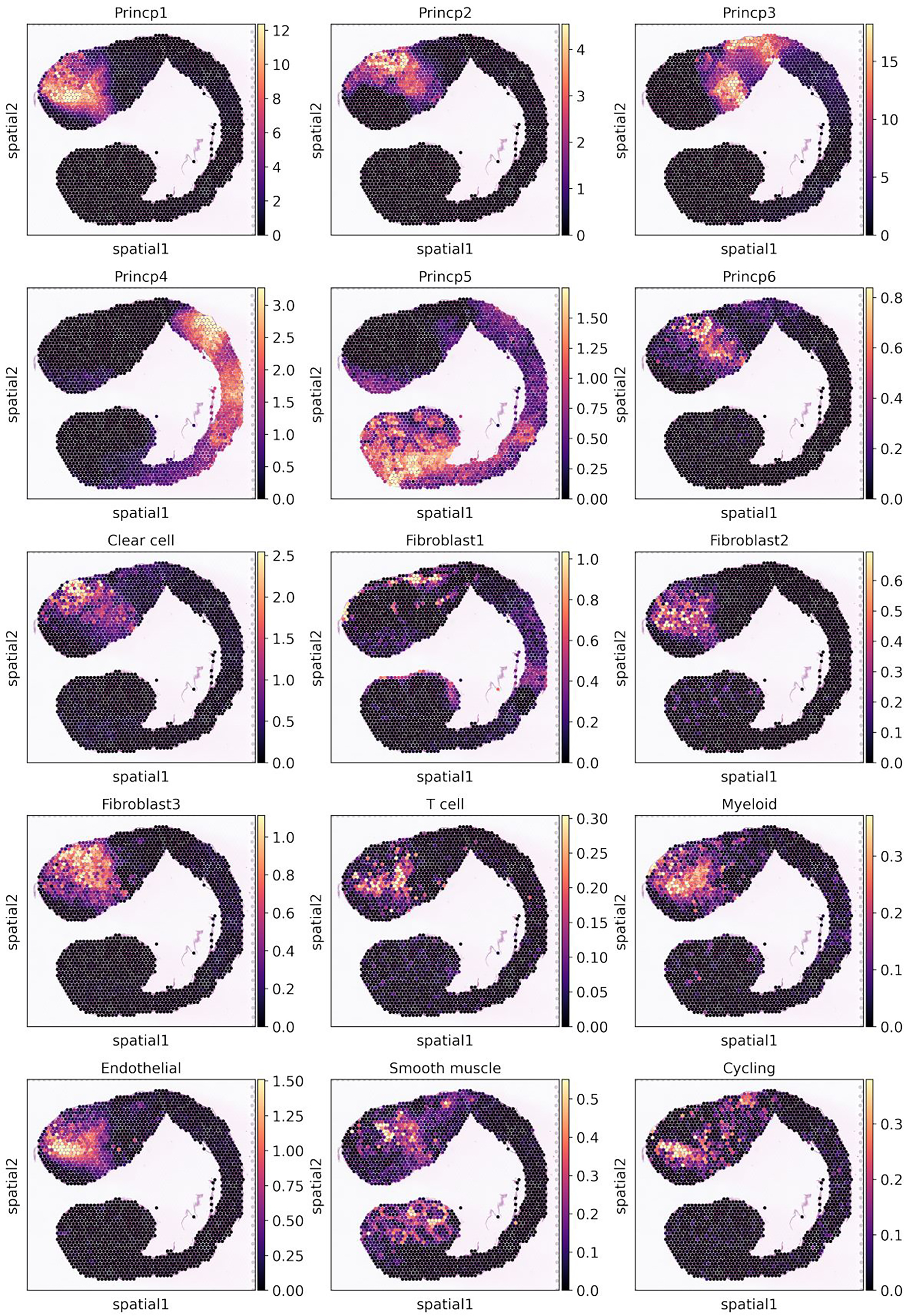

Supplement: Supplementary file 6 — Supplementary Material 6. Fig. S6 Mapping spatial data with scRNA-Seq cell type annotations by using Cell2location in the longitudinal spatial sample. Related to Figure 5. A) Mapping spatial data with scRNA-Seq cell type annotations of epididymal epithelium by using Cell2location. B) Mapping spatial data with scRNA-Seq cell type annotations of stromal cells by using Cell2location. C) Mapping spatial data with scRNA-Seq cell type annotations of all cells by using Cell2location. [file 12964_2024_1637_MOESM6_ESM.zip › Figure S6-2.jpg]

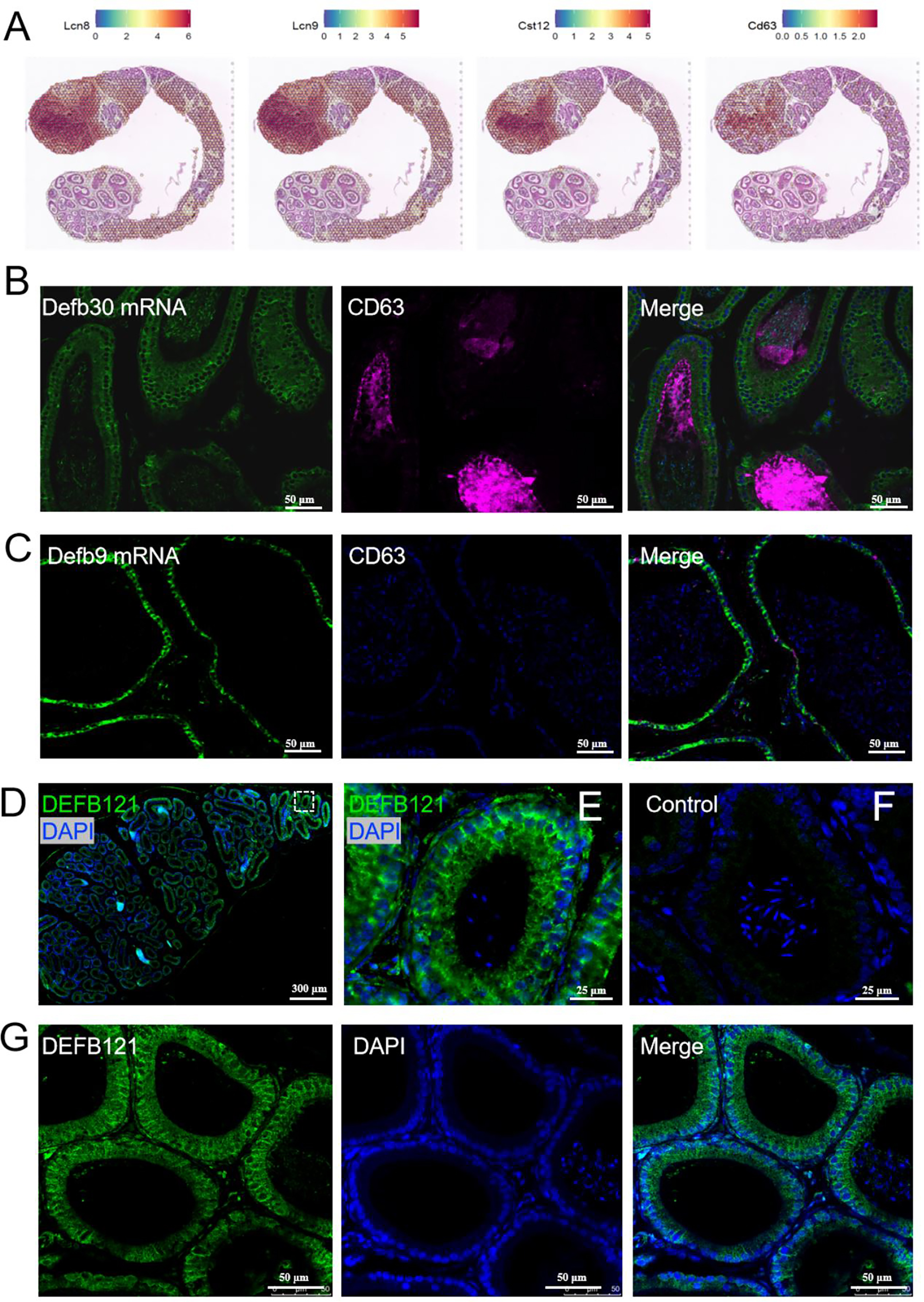

Supplement: Supplementary file 7 — Supplementary Material 7. Fig.S7 Beta-defensin gene showed region specificity in the mouse epididymal tissues. Related to Figure 6. A) Spatial gene expression features of lipocalin 8 (Lcn8), lipocalin 9 (Lcn9), Cst12, and Cd63 in the longitudinal spatial sample. B) Representative IF staining of CD63 and FISH staining of Defb30 mRNA in the corpus region of epididymis. Scale bar: 50 μm. C) Representative IF staining of CD63 and FISH staining of Defb9 mRNA in the cauda region of epididymis. Scale bar: 50 μm. D) Representative IF staining of DEFB121 in the caput epididymis. Scale bar: 300 μm. E) Enlarged view of Fig. S7D (white frame), showing DEFB121 is positive in principal cells. Scale bar: 25 μm. F) Negative control of DEFB121 IF staining (without primary antibody). Scale bar: 25 μm. G) Representative IF staining of DEFB121 in the posterior region of caput. Scale bar: 50 μm. [file 12964_2024_1637_MOESM7_ESM.jpg]

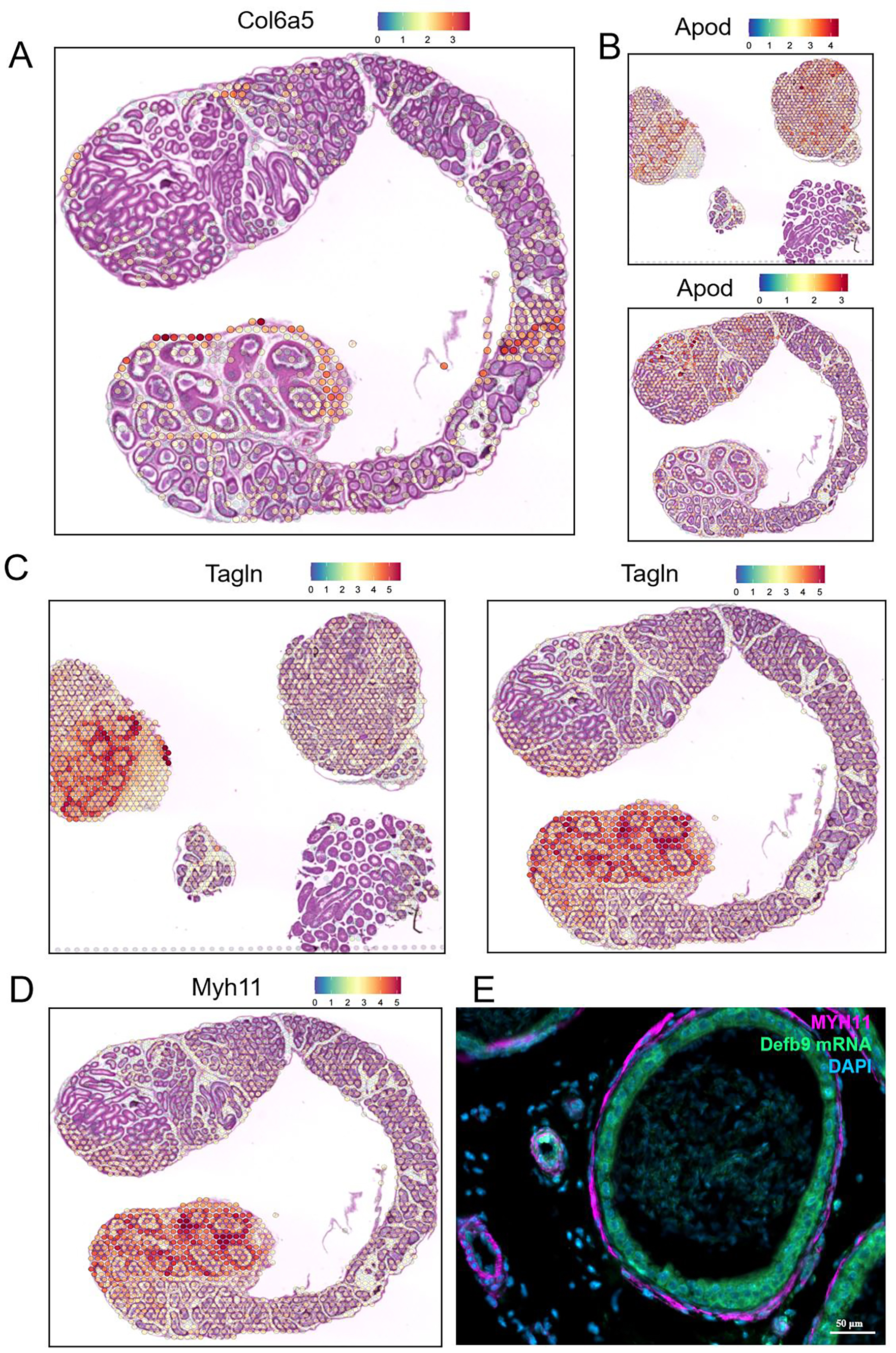

Supplement: Supplementary file 8 — Supplementary Material 8. Fig. S8 Gene expression profile of stromal cells in mouse epididymis. Related to Figure 7. A) Spatial gene expression feature of Col6a5 in longitudinal spatial sample. B) Spatial gene expression feature of Apod in crosscut and longitudinal spatial samples. C) Spatial gene expression feature of Tagln in crosscut and longitudinal spatial samples. D) Spatial gene expression feature of Myh11 in longitudinal spatial sample. E) Representative IF staining of MYH11 and FISH staining of Defb9 mRNA in the front-end region of caudal epididymis. [file 12964_2024_1637_MOESM8_ESM.jpg]
